# Trained autologous cytotoxic T-cells derived from PBMCs or splenocytes for immunotherapy of neuroblastoma

**DOI:** 10.3389/fimmu.2025.1546441

**Published:** 2025-06-09

**Authors:** Xiaofang Wu, Mousumi Basu, Sarah L. Wright, Samuel Li, Mikael Petrosyan, Marie V. Nelson, Alex I. Halpern, Douglas Shea, Mark Yarmakovich, Anthony D. Sandler

**Affiliations:** ^1^ The Joseph E. Robert Jr. Center for Surgical Care and The Sheikh Zayed Institute for Pediatric Surgical Innovation, Children’s National Hospital, George Washington University, Washington, DC, United States; ^2^ Perlmutter Cancer Center, New York University Grossman School of Medicine, New York, NY, United States

**Keywords:** neuroblastoma, training, autologous PBMCs, T cells, immunotherapy

## Abstract

**Background:**

Pediatric solid tumors, particularly neuroblastoma, present significant treatment challenges due to the limited efficacy of existing therapies. Adoptive immunotherapy, which involves transferring immune cells has shown clinical promise. Optimizing the preparation of immune cells *ex vivo* is critical to enhancing tumor immunity. This study introduces a novel method for improving the efficacy of autologous peripheral blood mononuclear cells (PBMCs) for neuroblastoma treatment.

**Methods:**

An IRB-approved protocol was used to collect tumor samples and PBMCs from eight patients undergoing neuroblastoma biopsy or resection. Primary tumor cells were isolated, cultured, and characterized using Phox2b and synaptophysin staining. Autologous PBMCs were co-cultured with irradiated tumor cells pre-treated with MYC inhibitors (I-BET726, JQ1) and a STING antagonist (C170) to enhance immunogenicity and train tumor-specific PBMCs. The immunogenicity and gene expression changes in treated tumor cells were assessed through multiplex ELISA and NanoString Tumor Signaling profiling. The phenotype and cytotoxicity of the trained PBMCs were evaluated by flow cytometry, IFN-γ ELISA, and IncuCyte assays.

**Results:**

Trained PBMCs primarily induced potent tumor cell cytotoxicity in patient-derived cellular products. In a preclinical neuroblastoma mouse model, similarly trained splenocytes demonstrated powerful efficacy, mirroring the findings in patient-derived PBMCs. This approach generates immunogenic tumor cells through modulation with small molecule inhibitors and radiation, enabling PBMCs or splenocytes to induce cytotoxic trained autologous tumor-specific T cells under controlled *in vitro* conditions. These trained PBMCs and splenocytes exhibit potent cytotoxicity against neuroblastoma, with significant therapeutic effects as an adoptive cellular immunotherapy *in vivo*.

**Conclusions:**

This study provides preliminary evidence supporting the efficacy of a personalized, PBMC-based immunotherapy for neuroblastoma. These findings highlight the potential for further development of this approach as a novel treatment strategy, paving the way for improved clinical outcomes in pediatric oncology.

## Introduction

Pediatric solid cancers, including neuroblastoma, remain a significant challenge despite advancements in chemotherapy and radiation therapy. Recurrent and refractory tumors are particularly resistant to standard therapy, resulting in survival rates of less than 10%. Consequently, there is an urgent need for innovative treatments, such as immunotherapy, to provide curative solutions.

Adoptive immunotherapy, which involves transferring immune cells like T cells and natural killer (NK) cells into patients, has shown promise in hematologic cancers such as leukemia, lymphoma, and myeloma (1–6). However, replicating this success in solid tumors has been difficult. Tumor-infiltrating lymphocytes (TILs) were initially explored for adoptive cell therapy in solid tumors, but the viability, expansion, and tumor-killing capabilities of these cells were limited (2, 3, 7–9). This highlights the need for more research into T cell-based therapies for pediatric solid tumors.

Chimeric antigen receptor (CAR)-T cell therapies have emerged as a groundbreaking and highly personalized approach to cancer treatment, offering remarkable success in hematologic malignancies and showing encouraging early results in neuroblastoma (10, 11). Their ability to redirect T cells toward tumor-specific antigens represents a major advancement in immunotherapy. However, despite this promise, broader clinical application in solid tumors like neuroblastoma remains limited by challenges such as restricted antigen availability (12, 13), insufficient T cell persistence (14–16), and potentially severe toxicities including cytokine release syndrome (CRS) (1, 17–21) and neurotoxicity (21–23). Another promising approach involves the use of γδ-T cells, which recognize unprocessed antigens and avoid graft-versus-host disease (GvHD) (24, 25). Despite their potential, *ex vivo* expansion of these cells remains a significant hurdle (26).

Peripheral blood mononuclear cells (PBMCs), which include T cells, NK cells, and antigen-presenting cells, offer a promising and easily accessible source for adoptive immunotherapy. Preclinical studies have shown that expanding or modifying PBMCs *ex vivo* can enhance their tumor-targeting abilities (27–29).

MYC proteins, including MYCN, c-MYC, and MYCL, play a critical role in tumorigenesis and are implicated in 70% of human cancers (30). These proteins help create an immune-privileged environment for tumors. Research has demonstrated that MYC inhibitors can enhance immunogenic pathways and make cancer cells more susceptible to immune attack (31–34). Inhibitors like I-BET726 and JQ1, which target bromodomain 4 (a key factor in MYC transcription), have shown promise in blocking MYC transcription and increasing cancer cell immunogenicity by suppressing PD-L1 expression (35, 36).

This research hypothesized that altering tumor cells *in vitro* would boost their immunogenicity, allowing them to train tumor-specific cytotoxic immune cells. Neuroblastoma tumor cells from pediatric patients were co-cultured with autologous PBMCs, resulting in potent cytotoxic capabilities. In preclinical mouse models, similarly trained splenocytes selectively targeted neuroblastoma tumors, suggesting a promising avenue for adoptive cell transfer therapy. Expanding tumor-reactive immune cells through co-culture with modified tumor cells provides a practical and clinically viable option for producing trained autologous PBMCs for adoptive cell therapy. These findings indicate the use of autologous PBMCs as a basis for patient-specific adoptive immunotherapy for neuroblastoma and potentially other solid tumors.

## Materials and methods

### Patient characteristics

Human tumor specimens were obtained from 8 patients diagnosed with neuroblastoma of which two were MYCN amplified. Diagnosis and staging were performed according to Children’s Oncology Group (COG) risk stratification and their clinical features are summarized in **Table 1**. Biopsies and blood draws were performed at the time of diagnosis as part of standard clinical care. All specimen collection for research purposes of this study was obtained after completion of appropriate consents and assents and was approved by the Institutional Review Board, Children’s National Hospital, Washington, DC (Pro00009692).

**Table 1:** Patient characteristics for human tumor specimens

Tumor Specimen	Age at time of tumor specimencollection (years)	Sex	INRG^†^Stage at diagnosis	MYCNamplification	Tumor specimen collection		Surviving
					Before chemo- therapy	After chemo- therapy	
HNB-1	2	F	M	No	Yes	Yes	Yes
HNB-2	3	M	M	Yes	Yes	–	Yes
HNB-3	8	F	L2	No	–	Yes	No
HNB-4	1	F	M	No	Yes	Yes	Yes
HNB-5	2	F	L1	No	–	Yes	Yes
HNB-6	3	F	M	Yes	–	Yes	Yes
HNB-7	2	M	M	No	–	Yes	Yes
HNB-8	2	F	M	No	–	Yes	Yes

^†^International Neuroblastoma Risk Group Staging.

### Cell line culture

The murine neuroblastoma Neuro2a cell line (Sigma, Missouri, USA) was cultured using EMEM supplemented with 2mM Glutamine, 1% Non-Essential Amino Acids (NEAA), 10% heat-inactivated fetal bovine serum (FBS, Sigma), 100 IU/mL penicillin and 100 µg/mL streptomycin. All media and supplements were purchased from Thermo Fisher Scientific (Waltham, Massachusetts, USA).

### Primary culture of human neuroblastoma cells

After washing with PBS, the tru-cut needle biopsy tumor tissue was minced into 1mm^3^ size and digested with 0.15mg/ml Liberase (Sigma, Burlington, MA) in 37°C water bath for 1h, and the digestion was stopped by adding 10% FBS (Gemini Bio, West Sacramento, California). The cells were centrifuged and washed twice. To eliminate the red blood cells, the pellet was treated with ACK lysing buffer (Thermofisher, Rockville, MD) according to the manufacturer’s instructions. The cells were then ready for culture. Cells were cultured with DMEM/F12 ham (Thermofisher) plus 10 ng/ml EGF (R&D system, Minneapolis, MN), 15 ng/ml bFGF (R&D system), 2% B27 supplement, and 1% penicillin/streptomycin (Thermofisher), 15% FBS (Gemini Bio Products), 1% non-essential amino acid (NEAA, Thermofisher), 1% sodium pyruvate (Thermofisher), plus 55 mM b-mercaptoethanol (Thermofisher). Cells were cultured in a 4.2ug/ml Laminin (Sigma) coated T25 flask in a 37°C, 5% CO2 tissue culture incubator.

### Isolation of peripheral blood mononuclear cells

Peripheral blood mononuclear cells (PBMCs) were isolated from peripheral blood draw of neuroblastoma patients using Ficoll-Hypaque (GE Healthcare, Uppsala, Sweden), as described by manufacturer’s protocol. After density gradient separation, PBMCs were placed in CryoStor^®^ cell cryopreservation media (Sigma-Aldrich, Saint Louis, MO) at 10^6^ cells/ml and cryopreserved in liquid nitrogen.

### Isolation of splenocytes and T cells from mouse spleen

Spleens were harvested from A/J mice (The Jackson Laboratory For Genomic Medicine, Farmington, CT), and splenocytes were isolated by homogenizing the spleen tissue, followed by lysing red blood cells with Gibco™ ACK Lysing Buffer (Thermo Fisher) according to the manufacturer’s protocol. This method typically yields about 3–4 x10^7^ splenocytes per single spleen. To isolate untouched T cells from splenocytes, we utilized the Pan T cell isolation kit II (Miltenyi Biotech, Gaithersburg, MD). This kit employs a negative selection process, wherein biotin-conjugated antibodies against CD11b, CD11c, CD19, CD45R (B220), CD49b (DX5), CD105, Anti-MHC-class II, and Ter-119-labeled mononuclear cells (Dendritic cells, NK cells, B cells, macrophages, monocytes) are separated from unlabeled T cells via a column in the presence of a magnetic field. For comprehensive methods, please refer to the manufacturer’s instructions.

### *Ex vivo* training of PBMCs or splenocytes

The tumor cells were treated with either 0.25 µM I-BET726 (Millipore Sigma, Massachusetts, USA), 0.25 µM JQ1 (Tocris, Minnesota, USA), or a combination of 0.25 µM BET, 0.25 µM JQ1, and a STING (stimulator of interferon genes protein antagonist—specifically, 1.5 µM C-170 (Cayman Chemical, Michigan, USA), 2 µM H-151 (Millipore Sigma, Burlington, MA), or 2 µM SN-011 (Cayman Chemical). Additionally, the STING protein agonist ADU-S100 (Chemietek, Indianapolis, IN) was combined with 0.25 µM BET/JQ1 at concentrations of 10 µg/ml and 20 µg/ml. The treatments were administered for 3 days in human primary neuroblastoma tumor cells and for 4 days in mouse Neuro2a cells. Following this treatment, the treated or untreated HNB and N2a tumor cells were irradiated at 65 Gray. Following irradiation, co-culture experiments were conducted by combining 2.5x10^6^ PBMC or splenocytes with 8x10^4^ human neuroblastoma cells or mouse neuro2a cells in a 24-well plate with 2ml of culture media. The culture media consisted of 2mM L-Glutamine (ThermoFisher, Waltham, MA), 10%FBS (Gemini Bio, West Sacramento, CA), 1% Insulin-Transferrin-Selenium (ITS) (ThermoFisher), 1% Penicillin-Streptomycin (ThermoFisher), 1% Sodium pyruvate (ThermoFisher), 1% Non-Essential Amino Acids (ThermoFisher), 20mM HEPES (ThermoFisher), 10ng/ml hEGF or mEGF (R&D syetem, Minneapolis, MN), 5ng/ml hFGF or mFGF (R&D system), 100ng/ml IL2 and 10ng/ml IL7 in RPMI1640 media (ThermoFisher). The co-cultures were maintained for a period of 7–10 days with media replenishment occurring every two to three days.

### Immunofluorescence staining

Primary human neuroblastoma cells were fixed in 4% paraformaldehyde (Sigma) for 15 min. Immunofluorescence staining was performed as previously described (35, 36). Primary antibodies directed against the following proteins were used: Phox2B (1:50, rabbit polyclonal, ThermoFisher), synaptophysin (20ug/ml, mouse monoclonal, R&D system) and nestin (clone 10C2,1:100, mouse monoclonal, ThermoFisher). Isotype-matched antibodies were used for negative controls. All fluorescent images were acquired with a Zeiss LSM 510 confocal microscope (Carl Zeiss MicroImaging, Thornwood, New York, USA). Images were taken at objectives of 20x and 40x, and the total magnification is 200x and 400x.

### *In vitro* IFNγ secretion assay

A total of 1×10^6^ human PBMCs or mouse splenocytes were cocultured with 5×10^4^ human neuroblastoma tumor cells or mouse Neuro2a cells in a 24 well plate for 48 hours. Supernatants were collected from triplicate wells, and IFNγ was assayed using the human or mouse uncoated IFNγ ELISA kit from Invitrogen (Carlsbad, California, USA). Readings were measured at 450 nm using the EnSpire 2300 Multilabel plate reader (Perkin Elmer, Waltham, Massachusetts, US).

### Multiplex cytokine/chemokine analysis

The concentrations of cytokines and chemokines were determined using the mouse 36-plex ProcartaPlex Panel (Thermo Scientific). Briefly, cell culture supernatant samples were mixed with antibody-linked polystyrene beads on a 96-well plate and incubated at room temperature (RT) for 2 hours. After washing, plates were incubated with biotinylated detection antibody for 30 min at room temperature (RT). The labeled beads were resuspended in streptavidin-PE for 30 min at RT, each sample was measured in duplicate along with standards (8-point dilutions) and the buffer control. Plates were read using a Luminex Bio-plex 200 system (Bio-Rad, Hercules, California, USA).

### Tumor cell cytotoxicity

The cytotoxicity of trained PBMC or splenocytes was determined by the Incucyte real time imaging system. Trained PBMCs/splenocytes or untrained PBMCs/splenocytes were incubated with target tumor cells in a 96-well plate at effector:target (E:T) ratios of 20:1 and 30:1. The cytotoxicity of PBMCs/splenocytes was determined by using the IncuCyte S3 Live Cell Analysis System. Briefly, human primary neuroblastoma cells or mouse Neuro2a cancer cells were labeled with 0.5uM Incucyte^®^ Cytolight Rapid Dyes (Sartorius) for 20 minutes and then incubated overnight at 37°C in a 96-well plate with a seeding density of 5000 cells per well. The following day, 50µl of Incucyte Caspase-3/7 dye was added to each well, followed by the addition of 1-1.5x10^5^ PBMCs or splenocytes per well. The plates were then transferred to the IncuCyte platform, where two images per well from triplicated wells were captured every 30 minutes for 21 hours using a 10× objective lens. Image analysis was performed using the IncuCyte™ 2021 B Software, wherein phase contrast was utilized for cell segmentation through mask application to exclude background cells. Additionally, an area filter excluded objects below 30 μm^2^, and background noise in the green and red channels was corrected using the Top-Hat method with a radius of 20 μm and a threshold of 2 corrected units.

### Mouse neuroblastoma therapeutic models

A total of 2 × 10^6^ Neuro2a cells were injected intraperitoneally (i.p.) into 6 weeks old A/J mice. After 3 days, 1x10^7^ splenocytes trained against BET/JQ1/C-170 treated N2a cells were administered to tumor-bearing mice by i.p. injection. The anti-tumor response was evaluated by following survival and monitoring tumor growth up to 4 weeks and 10 weeks. Laparotomy was performed to evaluate the tumor burden at those time points. In the 4 week therapeutic trial, several tissue systems were harvested from each mouse to evaluate autoimmune response using the nanostring nCounter mouse auto immune profiling. All the animal procedures were approved by the IACUC at Children’s National Hospital and are in accordance with the humane care of research animals. We used the ARRIVE1reporting guidelines (37).

### Nanostring

RNA extraction was conducted, and gene expression levels were quantified directly by measuring the corresponding mRNA counts in each sample using the nCounter human Tumor Signaling 360 Panel and mouse AutoImmune Profiling Panel (NanoString, Seattle, WA, USA). Detailed methodologies can be found in our prior publication (35, 36). Briefly, 100 ng of high-quality total RNA underwent hybridization with reporter probes followed by biotinylated capture probes at 65°C for 16–18 hours. Subsequently, the samples were processed in the nCounter Prep station and affixed to a cartridge. The cartridges were then analyzed using the nCounter Digital Analyzer optical scanner. Advanced immuneprofiling analysis was conducted using nSolver 4.0 analysis software along with the nCounter advanced analysis package (NanoString Technologies), which allowed for the identification of immune cell types. Genes were categorized into 14 immune cell types and 40 immune functions based on the manufacturer’s specifications.

### Statistics

The analysis of nanostring gene expression, including normalization, clustering, Pathview plots, and fold-changes, was conducted utilizing the Advanced Analysis Module within the nSolver™ Analysis Software version 4.0 by NanoString Technologies (NanoString Technologies, WA, USA), following our established protocols (35, 36). Initially, raw data for each sample underwent normalization to the geometric mean of housekeeping genes using the geNorm algorithm. Pathway scores were determined as the first principal component (PC) of the normalized expression of pathway genes. Cell type scores were centered to achieve a mean of 0, and since abundance estimates (cell type scores) were calculated in log2 scale, an increase of 1 corresponded to a doubling in abundance. All differentially expressed genes underwent KEGG term analysis, with significance determined at p < 0.05. To control the false discovery rate, the Benjamini-Yekutieli method was applied. The statistical analyses of nanostring data were performed using R v3.4.3 software (35).

Statistical significance for each set of experiments was determined using the unpaired 2-tailed Student’s t-test, with specific tests specified in the figure legends. Data are presented as the mean (± SD), with p < 0.05 considered statistically significant.

## Results

### Establishing and phenotyping primary human neuroblastoma cell lines

**Figure 1:**
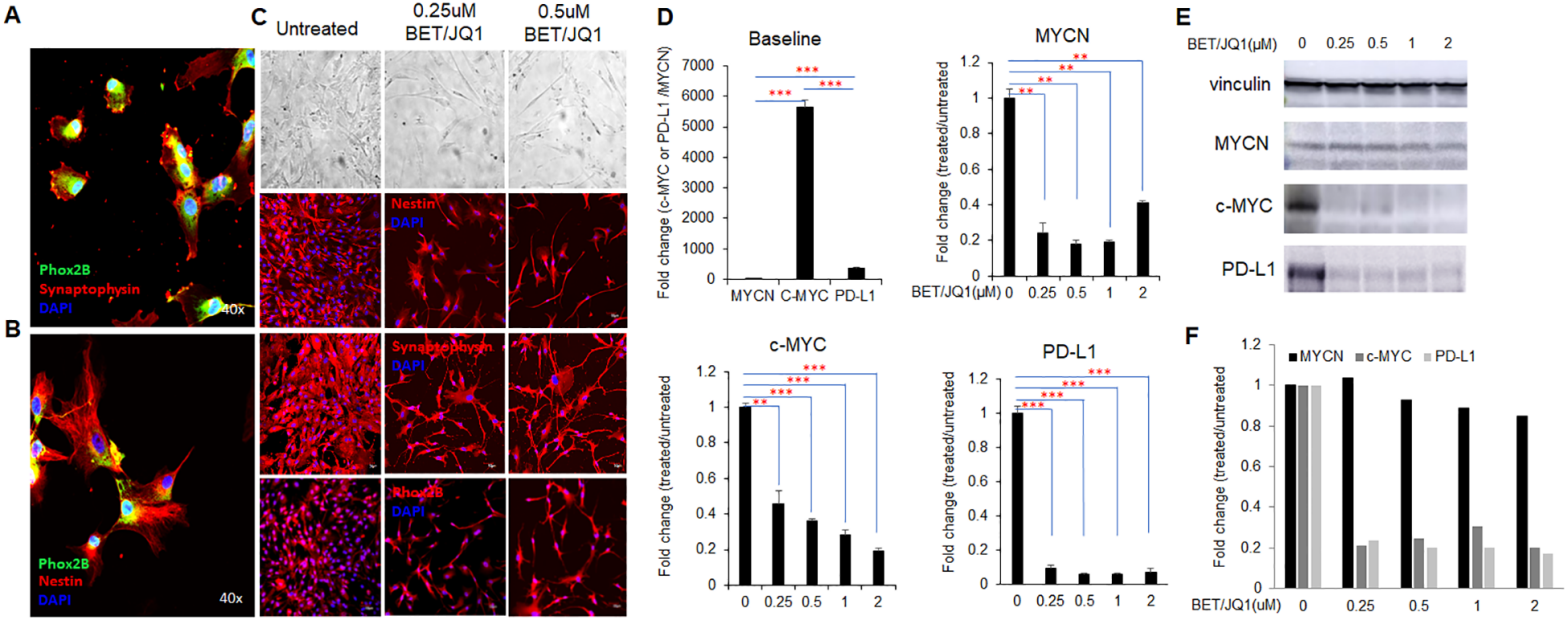
MYC inhibition promotes human neuroblastoma cell phenotypic differentiation and alters gene expression. **(A, B)** Primary culture of human neuroblastoma (HNB) with fluorescent confocal microscopy images of Phox2B, Synaptophysin, and Nestin staining, colors denoting each marker and total magnification at ×400. **(C)** bright-field and fluorescent microscopy images depicting morphological changes in HNB cells after 3 days of 0.25µM BET and 0.25µM JQ1 treatment, with original total magnification at ×200. Representative images are from the cells of the HNB-1 patient. **(D)** qRT-PCR analysis of MYCN, c-MYC, and PD-L1 mRNA expression in HNB cells treated with varying concentrations of BET and JQ1 for 3 days, using GAPDH as an internal control. HNE-1, HNE-2, and HNE-3 were used as individual qPCR samples. Results show mean score ± SD, with significance levels indicated (**p<0.01; ***p<0.001) determined by unpaired two-tailed Student’s t-test. **(E)** western blot results and **(F)** semi-quantification indicate protein expression of c-MYC, and PD-L1 in HNB-1 cells treated with the same drugs, with Vinculin as an internal control.

Human tumor specimens were obtained from 8 patients at Children’s National Hospital (CNH) diagnosed with neuroblastoma of which two were MYCN amplified. Diagnosis and staging were performed according to Children’s Oncology Group (COG) risk stratification and their clinical features are summarized in **Table 1** To characterize and verify primary cultured human neuroblastoma cells (HNB cells) isolated from 8 patient biopsies, we utilized immunohistochemical staining with PHOX2B and synaptophysin markers. PHOX2B, a highly specific marker for neuroblastic tumors, exhibited strong nuclear staining across all primary cultured human neuroblastoma cells (**Figures 1A**), while synaptophysin staining revealed clear cytoplasmic patterns, facilitating individual tumor cell identification (**Figure 1**). Additionally, the presence of the stem cell marker Nestin, associated with cancer cell aggressiveness and stemness, was observed in most primary neuroblastoma cells (**Figure 1**).

### MYC suppression with bromodomain inhibitors promotes neuroblastoma cell differentiation and alters gene expression

Previous studies have shown the effects of targeting Myc in mouse neuroblastoma cells, thus we wished to evaluate the effect on primary HNB tumor cell lines (35, 36). Morphological changes indicative of differentiation, such as increased neurite protrusions, were evident in cells treated with the MYC inhibitor I-BET726/JQ1 compared to untreated controls (**Figure 1**). We investigated the impact of I-BET726 and JQ1 on gene and protein expression in HNB cells over various treatment durations and concentrations via real-time qPCR analysis. Results revealed significant suppression of MYCN, C-MYC and PD-L1 with the combination treatment, particularly at 0.25 µM concentrations for 3 days (**Figure 1**). Western blot analysis confirmed that while C-MYC and PD-L1 were suppressed by the MYC inhibitor, MYCN remained unaffected (**Figure 1E**). This may be attributed to the patient’s MYCN non-amplified status, resulting in inherently low baseline MYCN levels. These findings replicate studies in murine cell line studies^26^ indicating the feasibility of bromodomain inhibition targeting MYC in primary human neuroblastoma cells.

### Bromodomain suppression of MYC in tumor cells induces immune activation of PBMCs that is enhanced by combination with a STING antagonist

**Figure 2:**
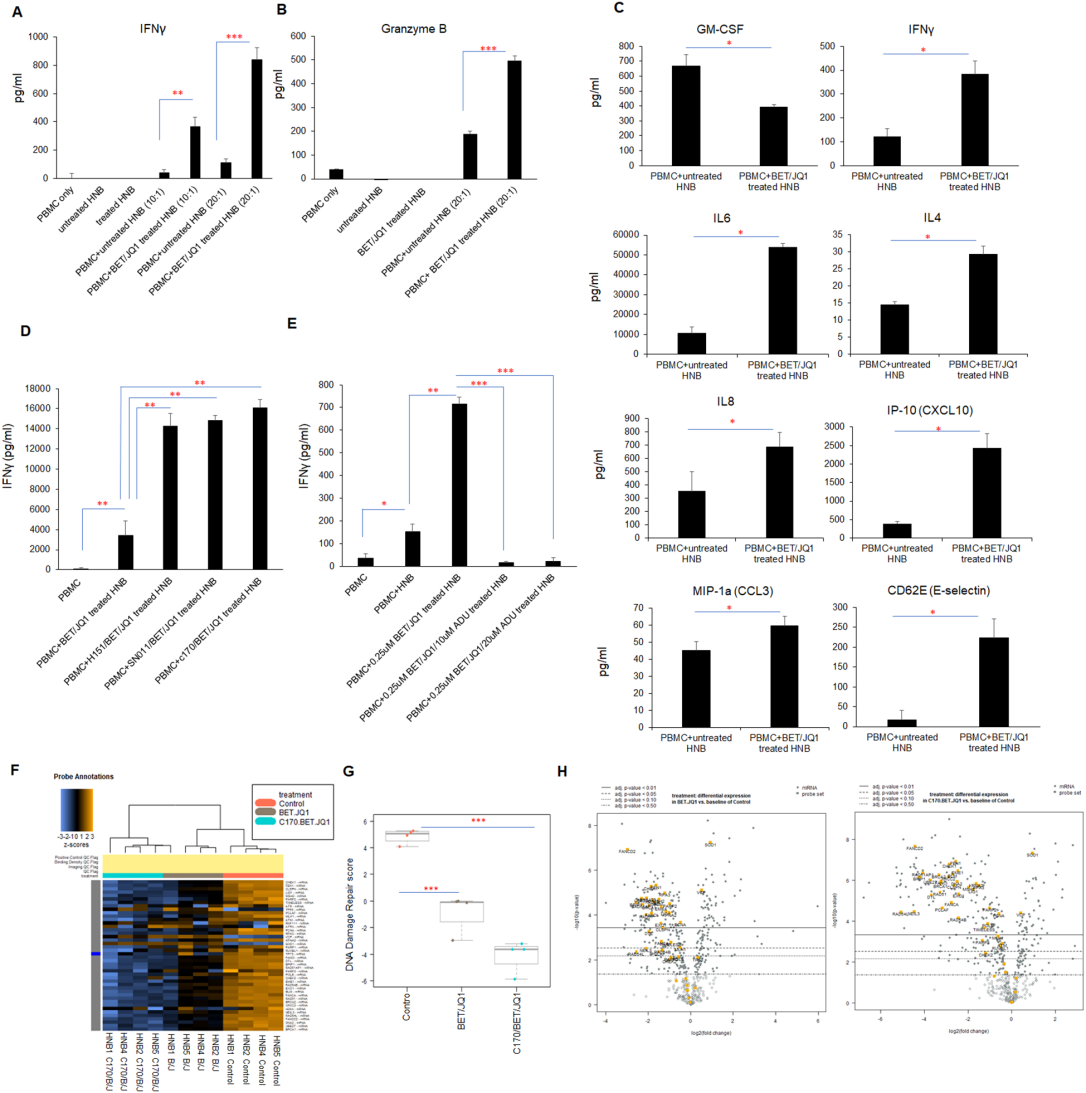
Inhibition of MYC expression in HNB cells enhances tumor cell immunogenicity. HNB-1, HNB-2 and HNB-3 cells were treated with BET (0.25 μM) and JQ1 (0.25 μM) for 3 days, followed by irradiation (60 Gy). Subsequently, treated (or untreated) HNB cells were co-cultured with autologous PBMCs for 48 hours at effector-to-target cell ratios of 10:1 or 20:1. **(A)** ELISA reveals a significant increase in IFNγ and **(B)** Granzyme B concentrations in the co-culture media with treated and irradiated HNB cells. **(C)** The co-cultured media were subjected to ProcartaPlex multiplex immunoassay analysis, demonstrating enhanced production of many pro-inflammatory cytokines and chemokines when PBMCs were cultured with treated and irradiated HNB cells. **(D, E)** ELISA results paradoxically revealed that Sting antagonists H151, SN011 and C170 enhanced IFNγ production during co-culture with immunogenic tumor cells, while STING agonist ADU-S100 inhibited IFNγ production in tumor cell/PBMCs reactions. **(F)** Modulation of cancer signaling pathways in human neuroblastoma cells through Myc targeting and Sting inhibition. HNB1–4 cells were treated with either 0.25 µM BET and 0.25 µM JQ1 or 1.5 µM C-170 and 0.25 µM BET/JQ1 for a duration of 3 days. Subsequently, gene expression profiles were analyzed using NanoString Human Cancer Signaling 360 Profiling. Heatmap of genes related to DNA damage repair in HNB cells, each row of the heat map is a single gene probe, and each column is a single sample. The bar at the top denotes treatment status, with orange=untreated control HNB cells (n=4), gray=BET/JQ1 treated HNB cells (n=4), blue=C-170/BET/JQ1 treated HNB cells (n=4). Yellow/brown indicates high gene expression; blue indicates low gene expression. Clear separation of gene expression is observed associated with treated and untreated cells. **(G)** Box plot of significantly decreased DNA damage repair pathway scores in treated versus untreated HNB cells, with median expression. Score is calculated in log2 scale, an increase of 1 on the vertical axis corresponds to a doubling in abundance. The horizontal black line on the box plot represents the median expression, and each symbol represents a single sample. The maximum and minimum expression level is represented by the upper and lower error bars, respectively. **(H)** Volcano plot displaying the differential expression (DE) of each gene related to DNA damage repair pathway. The left panel is showing the DE gene in BET/JQ1 treated HNB cells compared with untreated cells, and right panel is showing the DE gene in C-170/BET/JQ1 treated HNB cells versus control. Volcano plot displaying each gene’s -log10(p-value) and log2 fold change with the selected covariate. Highly statistically significant genes fall at the top of the plot above the horizontal lines, and highly differentially expressed genes fall to either side. Horizontal lines indicate various adjusted p-value thresholds. The most statistically significant genes are labeled yellow in the plot. Data are representative of three independent experiments. Results show mean score ± SD, with significance levels indicated (*p<0.05; **p<0.01; ***p<0.001) determined by unpaired two-tailed Student’s t-test.

To investigate the impact of MYC gene downregulation on tumor cell immunogenicity, primary HNB cell lines treated with 0.25µM I-BET726/JQ1, as well as untreated control tumor cells were irradiated and co-cultured with autologous PBMCs. After 48 hours, PBMCs produced significantly higher levels of IFNγ and Granzyme B when co-cultured with tumor cells treated with MYC inhibitors (**Figures 2A**). Additionally, we utilized ProcartaPlex multiplex immunoassay to analyze other cytokine/chemokine profiles with culture. A significant upregulation of seven out of twelve detectable cytokines was observed when PBMCs were co-cultured with MYC-inhibited primary HNB cells, including IFNγ, IL4, IL6, IL-8, MIP-1a (CCL3), IP-10 (CXCL-10), and CD62E (E-selectin). In contrast, GM-CSF was significantly downregulated (p < 0.05) (**Figure 2**). These findings underscore the immunogenic effects of MYC-inhibited tumor cells on PBMCs in co-culture.

Subsequently, we wished to determine if immunity was enhanced due to activation of stimulator of interferon response cGAMP interactor 1 (STING) pathways in the treated tumor cells. Western Blot analysis of treated tumor cells demonstrated activation and phosphorylation of STING (**Supplementary Figure 1**). To evaluate the role of STING in immune activation induced by treated cancer cells, we repeated the experiments in the presence of three STING inhibitors. Surprisingly, co-culturing PBMCs with tumor cells treated with BET/JQ1, in combination with STING pathway inhibitors (H151, SN011 and C-170) and irradiation, significantly and paradoxically boosted the production of IFNγ (**Figure 2**). Furthermore, the STING activator ADU-S100 effectively counteracted the activation effect induced by BET/JQ1 (**Figure 2**). These results show that the combination of BET/JQ1 treatment with a STING inhibitor and irradiation has a synergistic effect of enhancing HNB tumor cell immunogenicity. Also of clinical interest, we observed that PBMCs collected from patients before chemotherapy exhibited a more robust response to BET/JQ1/C-170-treated tumor cells compared to those collected after chemotherapy (**Supplementary Figure 2**). This finding highlights the necessity of collecting PBMCs prior to chemotherapy when attempting to optimize *ex vivo* immunotherapeutic approaches.

### Targeting Myc suppresses expression of DNA damage repair genes associated with enhanced cellular immunity

To further elucidate the changes by which MYC suppression effects human neuroblastoma tumor cells, we conducted a comparative analysis using NanoString Human Cancer Signaling 360 Profiling. We examined MYC-targeted HNB cells alongside untreated HNB cells, both with and without STING inhibition and observed notable changes in various cellular pathways. In our analysis (n=4 HNB cell lines), represented in **Supplementary Figure 3A**, we observed significant downregulation in signaling scores related to the MYC pathway, DNA damage repair, cell cycle, apoptosis and stemness among several other pathways. Conversely, scores related to inflammation, interferon response, autophagy and glucose metabolism were significantly upregulated. Specifically focusing on the DNA damage repair pathway, blocking MYCN with BET/JQ1 and inhibiting STING with C170 led to decreased expression of genes involved in multiple DNA damage repair pathways. This is evidenced by the heatmap in **Figure 2**, showing gene changes across samples (n=4 in each group), and further supported by the significant downregulation of the DNA damage repair score depicted in the box plot in **Figure 2**. Additionally, the volcano plot in **Figure 2** illustrates that the combination of C170 with BET/JQ1 resulted in even greater inhibition of DNA damage repair genes compared to BET/JQ1 alone. Defects in these pathways can trigger immunostimulation by leading to tumor cell death and producing neoantigens, promoting anti-tumor immunity (38–41). The downregulated genes associated with DNA damage repair in the BET/JQ1 versus control group are listed in **Table 2**, while those in the C170/BET/JQ1 groups are listed in **Table 3**. Taken together, the findings suggest that MYC suppression, particularly in conjunction with C170 induces tumor cell immunity by disrupting multiple DNA damage repair pathways among other potential mechanisms in human neuroblastoma tumor cells.

**Table 2:** Top statistically significantly down-regulated genes in DNA damage repair in BET/JQ1 treated HNB cells compared with untreated control (p value<0.05).

Probe. Label	Linear fold change	P-value	Probe. Annotation
FANCD2	-7.81	1.17E-07	p53 Signaling; DNA Damage Repair
BRIP1	-5.95	2.83E-05	DNA Damage Repair; Cell Cycle
RAD54L	-5.85	0.0045	DNA Damage Repair
UBE2T	-4.98	1.83E-05	DNA Damage Repair
RAD51AP1	-4.76	3.39E-05	DNA Damage Repair
NEIL3	-4.29	0.0031	DNA Damage Repair
RAD51	-4.27	9.82E-05	DNA Damage Repair; Cell Cycle
BRCA2	-4.20	0.00002	DNA Damage Repair
DTL	-3.89	0.00055	DNA Damage Repair
BRCA1	-3.86	0.00004	DNA Damage Repair; Androgen Signaling
CHEK1	-3.62	5.32E-06	p53 Signaling; DNA Damage Repair
FANCI	-3.58	8.39E-05	p53 Signaling; DNA Damage Repair
XRCC2	-3.51	2.08E-05	DNA Damage Repair
DNA2	-3.30	2.64E-05	Immortality & Stemness; DNA Damage Repair; Cell Cycle
FEN1	-3.09	4.65E-06	Immortality & Stemness; DNA Damage Repair; Cell Cycle
BLM	-2.86	2.64E-05	DNA Damage Repair
PCLAF	-2.84	0.005	DNA Damage Repair
MSH2	-2.81	1.93E-05	DNA Damage Repair
EXO1	-2.63	0.0002	DNA Damage Repair; Cell Cycle
EME1	-2.60	4.32E-05	DNA Damage Repair
FANCA	-2.59	0.0015	DNA Damage Repair
RAD54B	-2.51	0.0037	DNA Damage Repair
CLSPN	-2.49	0.0004	DNA Damage Repair; Cell Cycle
RPA3	-2.32	1.04E-05	Immortality & Stemness; DNA Damage Repair; Cell Cycle
LIG1	-2.23	0.00007	Immortality & Stemness; DNA Damage Repair; Cell Cycle
KPNA2	-1.99	8.16E-05	Interferon Response; Estrogen Signaling; DNA Damage Repair
CHEK2	-1.98	0.0099	DNA Damage Repair
PARP2	-1.96	3.17E-05	DNA Damage Repair
TIMELESS	-1.90	0.0017	DNA Damage Repair
PARP3	-1.89	0.008	DNA Damage Repair
POLE	-1.49	0.007	Immortality & Stemness; DNA Damage Repair; Cell Cycle
MLH1	-1.40	0.0028	DNA Damage Repair

**Table 3:** Top statistically significantly down-regulated genes in DNA damage repair in C170/BET/JQ1 treated HNB cells compared with untreated control (p value<0.05).

Probe. Label	Linear fold change	P-value	Probe. Annotation
RAD54L	-33.56	3.72E-05	DNA Damage Repair
NEIL3	-22.22	3.62E-05	DNA Damage Repair
FANCD2	-21.55	2.24E-08	p53 Signaling; DNA Damage Repair
BRIP1	-18.59	7.08E-07	DNA Damage Repair; Cell Cycle
RAD51AP1	-16.64	4.52E-07	DNA Damage Repair
UBE2T	-13.18	1.02E-06	DNA Damage Repair
DTL	-12.82	5.44E-06	DNA Damage Repair
BRCA2	-10.06	5.74E-07	DNA Damage Repair
RAD51	-9.71	3.93E-06	DNA Damage Repair; Cell Cycle
BRCA1	-9.62	1.61E-06	DNA Damage Repair; Androgen Signaling
FANCI	-9.43	1.13E-06	p53 Signaling; DNA Damage Repair
PCLAF	-9.09	2.38E-05	DNA Damage Repair
XRCC2	-7.46	7.08E-07	DNA Damage Repair
CHEK1	-7.19	1.67E-07	p53 Signaling; DNA Damage Repair
FANCA	-6.80	1.29E-05	DNA Damage Repair
BLM	-6.54	5.13E-07	DNA Damage Repair
CLSPN	-6.21	1.84E-06	DNA Damage Repair; Cell Cycle
FEN1	-5.88	1.21E-07	Immortality & Stemness; DNA Damage Repair; Cell Cycle
DNA2	-5.65	1.22E-06	Immortality & Stemness; DNA Damage Repair; Cell Cycle
EME1	-5.52	3.5E-07	DNA Damage Repair
RAD54B	-5.43	7.13E-05	DNA Damage Repair
EXO1	-5.38	4.81E-06	DNA Damage Repair; Cell Cycle
MSH2	-3.88	2.29E-06	DNA Damage Repair
LIG1	-3.36	2.98E-06	Immortality & Stemness; DNA Damage Repair; Cell Cycle
KPNA2	-3.10	1.49E-06	Interferon Response; Estrogen Signaling; DNA Damage Repair
RPA3	-2.82	1.89E-06	Immortality & Stemness; DNA Damage Repair; Cell Cycle
PARP3	-2.53	0.0009	DNA Damage Repair
TIMELESS	-2.38	0.0002	DNA Damage Repair
CHEK2	-2.28	0.0037	DNA Damage Repair
POLE	-2.13	0.0001	Immortality & Stemness; DNA Damage Repair; Cell Cycle
PARP2	-2.02	2.34E-05	DNA Damage Repair
H2AX	-1.73	0.05	DNA Damage Repair
MLH1	-1.55	0.0006	DNA Damage Repair
PCNA	-1.50	4.39E-05	Immortality & Stemness; DNA Damage Repair; Cell Cycle
RUVBL1	-1.42	0.0018	Wnt Signaling; Immortality & Stemness; DNA Damage Repair; Cell Cycle
ATM	-1.35	0.0013	Senescence; p53 Signaling; Epigenetic & Transcriptional Regulation; DNA Damage Repair
PARP1	-1.26	0.013	NF-kB Signaling; DNA Damage Repair
ATR	-1.22	0.033	p53 Signaling; DNA Damage Repair

### PBMCs trained with treated immunogenic tumor cells produce potent cytotoxic activity

**Figure 3:**
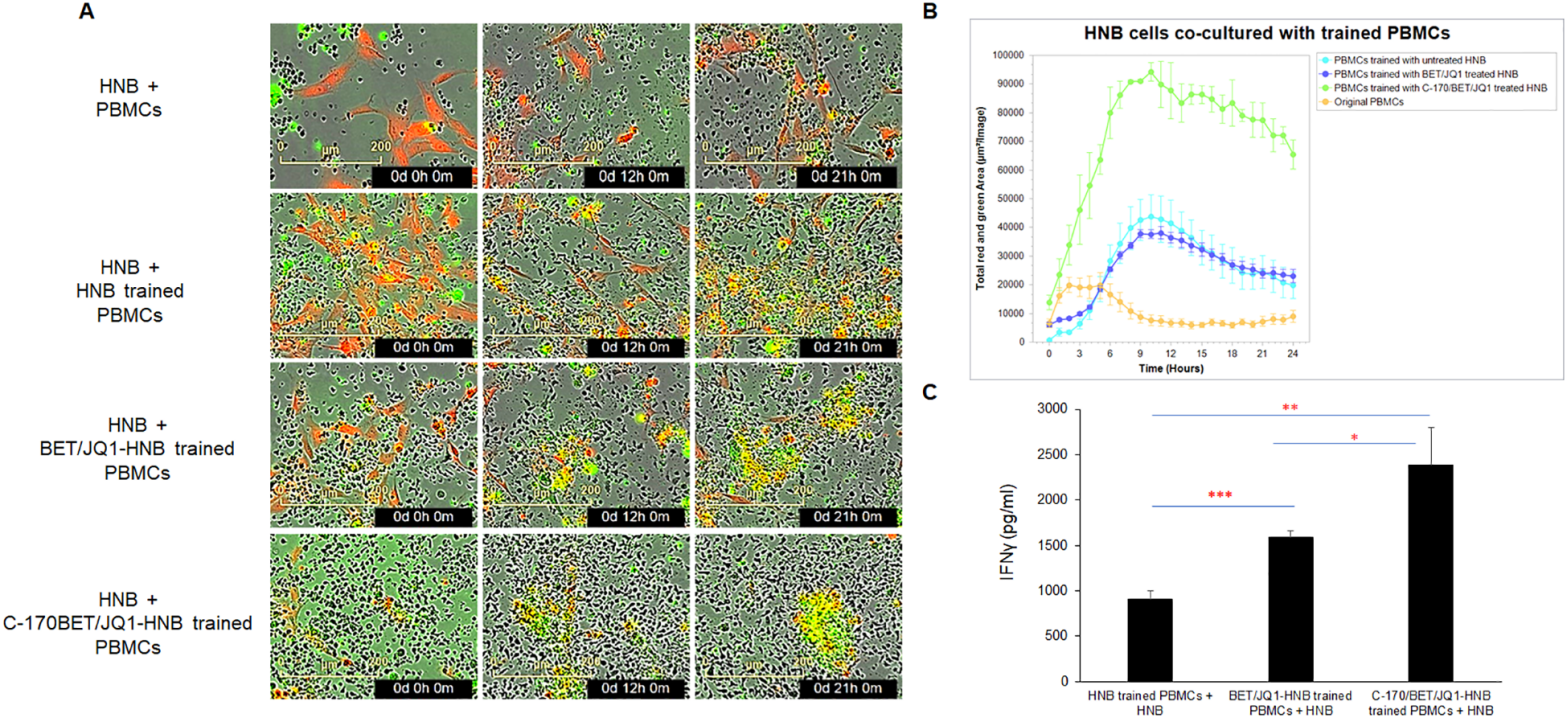
Trained PBMCs generate potent cytotoxic activity via co-culture with irradiated BET/JQ1/C170 treated tumor cells, assessed by live-cell imaging. **(A)** IncuCyte Live-Cell Analysis System was used to assess T cell cytotoxicity. PBMCs were trained with irradiated untreated HNB cells, HNB cells treated with 0.25µM BET/JQ1, or with 1.5 µM C-170 and 0.25 µM BET/JQ1 for 7 days. Subsequently, HNB cancer cells were co-cultured with untrained PBMCs or trained PBMCs at a ratio of 1:30 in 96-well plates. HNB tumor cells were stained with CytoLight Red dye, and apoptotic cells are marked by Caspase 3/7 Green. The yellow color indicates apoptotic tumor cells undergoing cytotoxicity. Representative time-lapse images captured by the IncuCyte system showing target cell lysis by cytotoxic T cells over a period of 21 hours. No viable tumor cells seen in HNB co-cultured with C-170/Bet/JQ1 trained PBMCs. **(B)** Quantification of target cell death over time, indicating the cytotoxic activity of T cells against target cells. Cells from patients HNB-1, 2, 4, 5, and 6 were all analyzed using the Incucyte assay. The results presented here are from HNB-2, with similar results noted in each cell line. **(C)** Quantification of IFNγ production from HNB tumor cell trained PBMCs by ELISA. Data are representative of three independent experiments. Cells from patient HNB-1,2,and 5 were used in this section. Results are presented as mean ± standard error of the mean from three independent experiments. Statistical significance was determined using unpaired two-tailed Student’s t-test (*p < 0.05, **p < 0.01, ***p < 0.001).

To assess the functional interactions between cancer cells and trained human PBMCs, we conducted experiments using the IncuCyte^®^ live-cell analysis system to evaluate cancer cell trained PBMCs from patient samples. Initially, PBMCs were co-cultured (trained) with untreated HNB cells, HNB cells treated with 0.25µM BET/JQ1, or HNB cells treated with 1.5 µM C-170 and 0.25 µM BET/JQ1 for either 7 or 14 days. Subsequently, HNB cancer cells were co-cultured with untrained PBMCs (serving as a control) or trained PBMCs in 96-well plates. Real-time cytotoxicity analysis was performed using the IncuCyte^®^ imaging system, monitoring tumor cell death with caspase 3 and 7 activation over 21 hours. We examined cytotoxicity by evaluating the ratio of effector (E) cells to target (T) cells at 20:1 or 30:1 as well as different training regimens: a single round for 7 days versus two rounds for 14 days. Our results indicate that the most effective and potent tumor cell killing was achieved with an E:T ratio of 30:1 when tumor cells were treated with a combination of C-170/BET/JQ1 for 7 days (**Figure 3A**, **Supplementary Video**). In addition, co-culturing PBMCs with tumor cells treated with BET/JQ1 combined with C-170 significantly boosted the production of IFNγ (**Figure 3**).

These cumulative findings demonstrate that PBMCs, cultured and trained *ex vivo* with autologous tumor cells that have been treated and modified to enhance immunogenicity, can generate potent autologous cytotoxic activity against the original untreated tumor cells. In order to test the trained PBMCs, we wished to recapitulate these findings in mouse studies in order to test their therapeutic efficacy in pre-clinical models of established neuroblastoma tumors.

### Mouse Neuro2a tumor cells irradiated and treated with Myc inhibitors and a STING antagonist (BET/JQ1/C-170) undergo similar phenotypic changes to human cancer cell lines and induce potent immune activation of splenocytes

**Figure 4:**
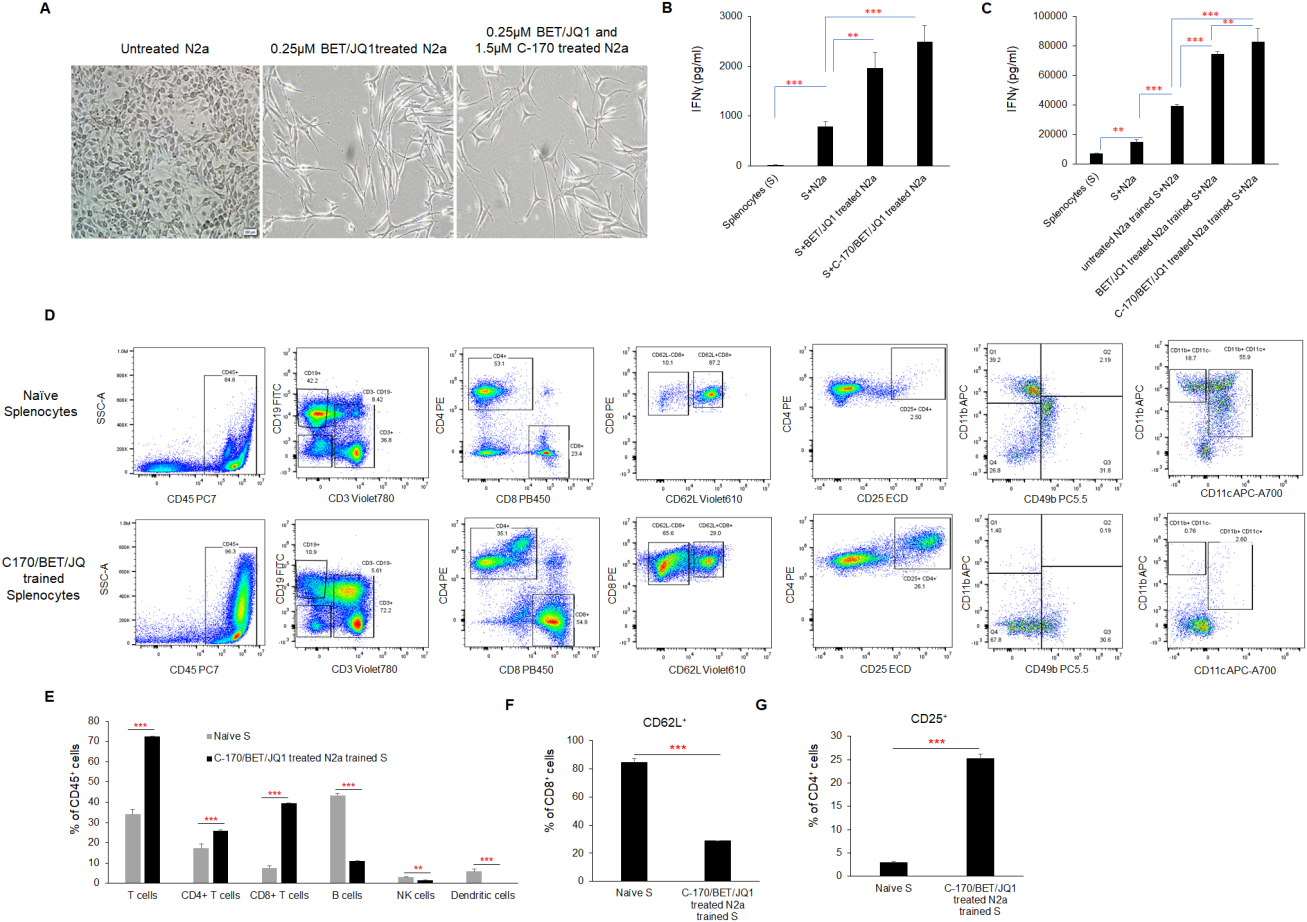
Phenotypic changes and immune activation induced by irradiated, BET/JQ1/C-170 treated mouse Neuro2a (N2a) tumor cells **(A)** Bright-field microscopy images depict morphological alterations in N2a cells following treatment with BET/JQ1, with original total magnification at ×200. **(B)** IFNγ production from splenocytes in the tumor cell-splenocyte reaction was assessed. To induce antigen exposure in mouse splenocytes, 2×10^6^ irradiated N2a cells (60Gy) and 100µg anti-CTLA-4 antibody were administered intraperitoneally to mice. After 7 days, antigen-exposed splenocytes (AS) were harvested and subjected to co-culture. ELISA results revealed a significant increase in IFNγ concentrations in the media when splenocytes were cocultured with treated N2a cells for 48h. **(C)** Assessment of IFNγ production from N2a tumor cell trained mouse splenocytes. Antigen exposed splenocytes were trained over 7 days with irradiated wild-type N2a cells, N2a cells treated with 0.25µM BET/JQ1, or N2a cells treated with 1.5 µM C-170 in combination with 0.25 µM BET/JQ1. Following training, wild-type N2a cancer cells were co-cultured with either untrained or trained splenocytes for 48 hours at a 1:20 ratio. IFNγ production in the culture media was quantified via ELISA assays. Results indicate a significant increase in IFNγ concentration when splenocytes were co-cultured with splenocytes trained with C-170/BET/JQ1-treated N2a cells. (S: splenocytes). **(D)** The flow cytometry analysis of the splenocytes trained with treated N2a tumor cells. Splenocytes were trained over 7 days with irradiated wild-type N2a cells, N2a cells treated with 0.25µM BET/JQ1, or N2a cells treated with 1.5 µM C-170 in combination with 0.25 µM BET/JQ1. Following training, splenocytes were stained with specific mAbs and analyzed by flow cytometry. The relevant isotype control sample was set as negative control (data not shown). Gating strategy used to analyze markers related to different types of immune cells. CD45^+^ cells: Lymphocytes; CD3^+^ cells: T cells; CD8^+^ CD3^+^ cells: Cytotoxic T cells; CD4^+^CD3^+^ Cells: T help/reg cells; CD8^+^CD62L^+^ cells: Effector and antigen-specific T cells, CD4^+^CD25^+^ cells: T regulatory cells (Tregs); CD49^+^ cells: NK cells, CD11b^+^CD11C^+^ cells: Dendritic cells. **(E)** The proportions of T cells, B cells, NK cells, and dendritic cells within the total lymphocyte population, along with the percentage of CD62L+ cells among CD8+ T cells **(F)** and CD25+ cells among CD4+ T cells **(G)**, were compared between C170/BET/JQ1-trained splenocytes and naïve splenocytes. Data are representative of three independent experiments. The bars represent means ± SD. Statistical significance was determined using unpaired two-tailed Student’s t-test (**p < 0.01, ***p < 0.001), (n=3 tests for each group).

To validate our human neuroblastoma tumor cell findings, we replicated the study using mouse Neuro2A (N2a) neuroblastoma cells. We found that N2a cells responded similarly to BET and JQ1 treatment, alone or in combination with C-170, mirroring the phenotypic changes seen in HNB cells (**Figure 4**). We induced antigen exposure in mouse splenocytes by injecting 2×10^6^ irradiated N2a cells subcutaneously and anti-CTLA-4 antibody intraperitoneally (i.p.) into mice. After 7 days, we harvested these Ag exposed splenocytes and initiated co-culture training. Over 7 days, splenocytes were trained with wild-type N2a cells, N2a cells treated with 0.25µM BET/JQ1, or N2a cells treated with 1.5 µM C-170 plus 0.25 µM BET/JQ1. Following training, we co-cultured wild-type N2a cancer cells with either untrained or trained splenocytes for 48 hours and quantified IFNγ production via ELISA assays. Our findings revealed a significant enhancement in IFNγ production when N2a tumor cells were co-cultured with splenocytes trained with BET/JQ1, with an even greater enhancement observed when combined with C-170 (**Figure 4B**).

### CD8 and CD4 effector T cells are primed from splenocytes trained with treated N2a tumor cells and are cytotoxic to wild type N2a tumor cells following training

To characterize the phenotype of trained splenocytes, we performed flow cytometry to analyze various immune cell populations (**Figures 4D–G**; **Supplementary Figure 4**). The analysis revealed a significant increase in the frequencies of CD3^+^ T cells (from 34% to 72%), CD8^+^ T cells (from 7% to 40%), and CD4^+^ T cells (from 17% to 25%) within the CD45^+^ population, while CD19^+^ B cells (from 43% to 11%), CD49b^+^ NK cells (from 3% to 1.6%), and CD11b^+^CD11c^+^ dendritic cells (from 5% to 0.2%) were markedly reduced (**Figure 4D**) in trained splenocytes compared to naïve, untrained splenocytes. Notably, the proportion of CD8^+^CD62L^+^ cells decreased from approximately 84% to 29% in trained splenocytes (**Figure 4F**), suggesting a shift toward an effector phenotype characterized by CD62L downregulation in response to prolonged antigen exposure (33, 34). Additionally, CD25^+^CD4^+^ regulatory T cells were significantly elevated in trained splenocytes compared to naïve counterparts (**Figure 4G**). A similar trend in each immune cell population across the different training groups is shown in **Supplementary Figure 4**. These findings indicate that post *ex vivo* training with treated immunogenic tumor cells, splenocytes undergo significant changes in their phenotypic composition, with expansion of an activated, primed effector T cell phenotype.

**Figure 5:**
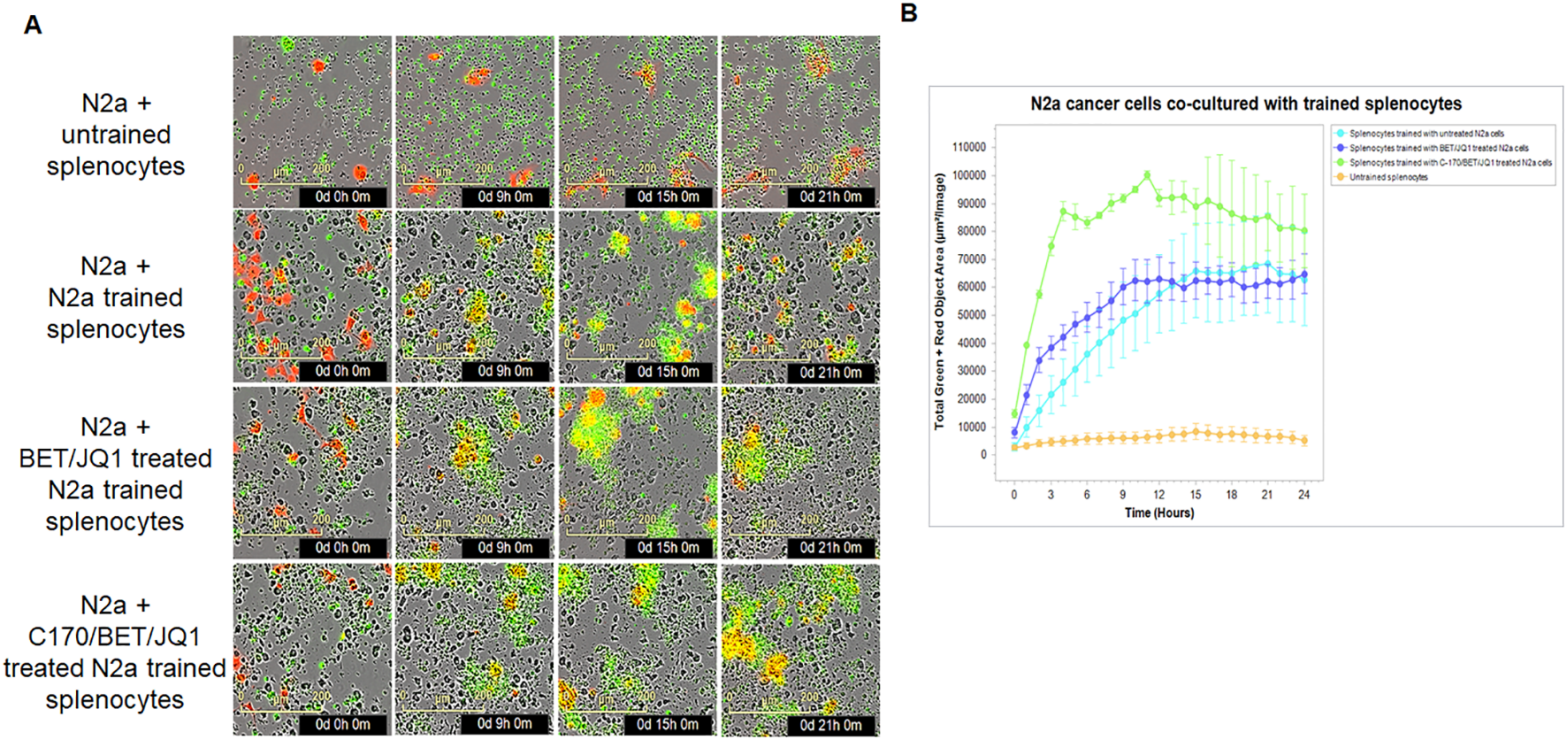
Tumor-trained splenocytes induce cytotoxic immune cells. **(A)** IncuCyte Live-Cell Analysis System was used to assess tumor cell cytotoxicity. Ag exposed splenocytes were trained with irradiated untreated N2a cells, N2a cells treated with 0.25µM BET/JQ1, or with 1.5 µM C-170 and 0.25 µM BET/JQ1 for 7 days. Subsequently, N2a cancer cells were co-cultured with untrained splenocytes or trained splenocytes at a ratio of 1:30 in 96-well plates. N2a tumor cells were stained with CytoLight Red dye, and apoptotic cells are marked by Caspase 3/7 Green. The yellow color indicates apoptotic tumor cells undergoing cytotoxicity. Representative time-lapse images captured by the IncuCyte system showing target N2a cell lysis by cytotoxic T cells over a period of 21 hours. **(B)** Quantification of target N2a cell death over time, indicating the cytotoxic activity of splenocytes against target cells.

We evaluated cytotoxicity of these trained cells in the syngeneic mouse N2a tumor using the IncuCyte system. Remarkably, our results mirrored those obtained in the human PBMC experiments. Although training of splenocytes against all the variations of treated tumor cells were cytotoxic compared to untrained splenocytes, those trained against BET/JQ1/C-170 treated N2a cells showed the greatest cytotoxicity to wild-type untreated N2a tumor cells (**Figure 5A**).

### Tumor trained splenocytes induce tumor regression in a murine neuroblastoma model following adoptive cell therapy

**Figure 6:**
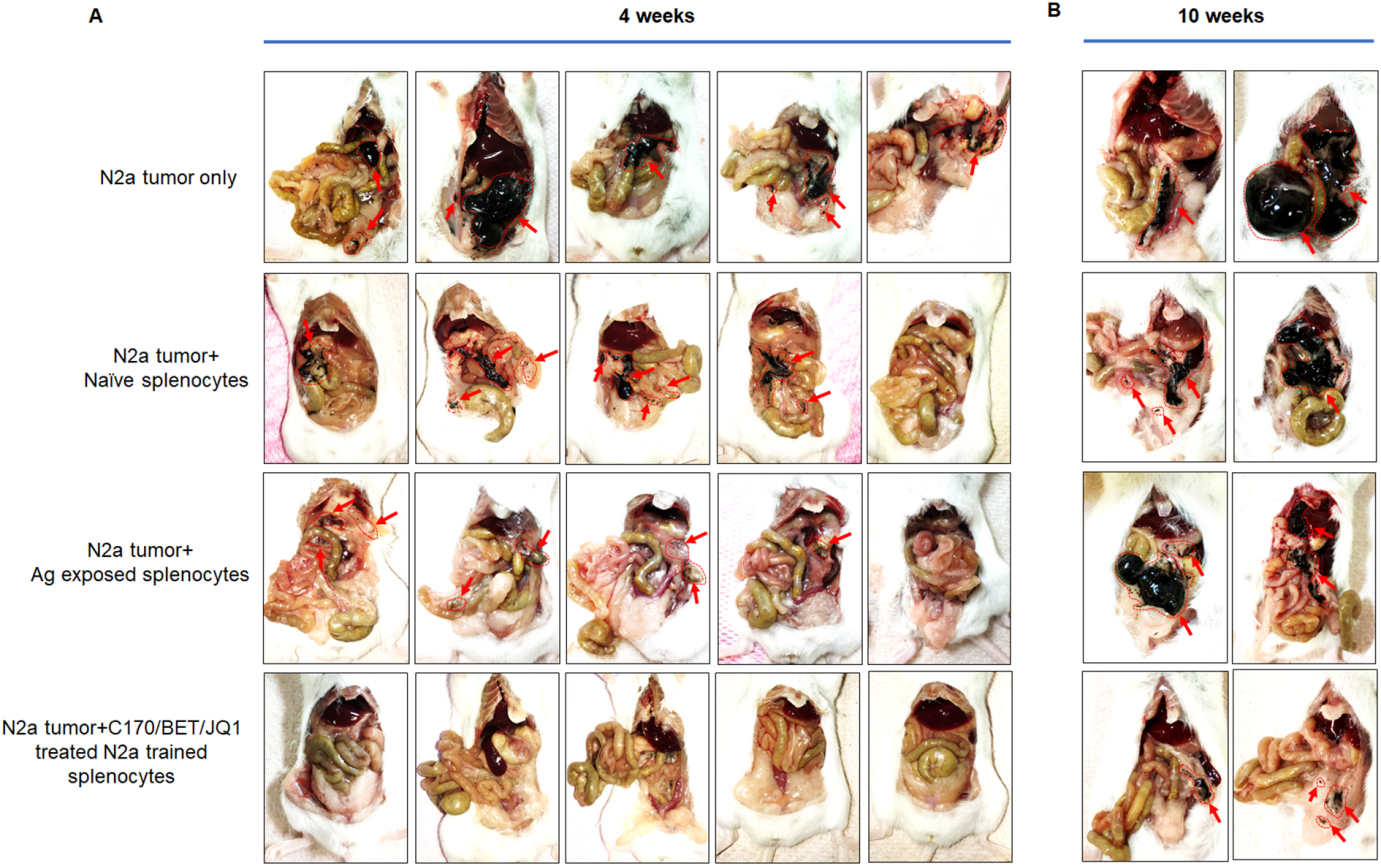
Tumor-trained splenocytes lead to regression of tumors in a mouse model of neuroblastoma. Antigen exposure splenocytes were trained over 7 days with irradiated N2a cells treated with 1.5 µM C-170 in combination with 0.25 µM BET/JQ1. Following the training period, mice were intraperitoneally administered with 2x10^6^ N2a cells along with 10^7^ trained or untrained splenocytes. Tumor growth was compared at 4 weeks **(A)** and 10 weeks **(B)** post-treatment. The findings revealed that all seven mice injected only with tumor cells developed tumors, along with six out of seven mice injected with tumor cells and either naïve splenocytes or splenocytes from Ag exposed mice. None of the mice injected with trained splenocytes developed tumors at 4 weeks, and tumor growth was remarkably repressed at 10 weeks following a single infusion of immune-cells. Red arrows and red dot circle points to tumor.

The therapeutic efficacy of trained tumor specific splenocytes was assessed in a mouse neuroblastoma model. Trained autologous splenocytes derived from A/J mouse were trained against either BET/JQ1 or BET/JQ1/C-170 treated or untreated N2a cells for a duration of 7 days as described above. Following the training period, mice were intraperitoneally administered with 2x10^6^ N2a cells along with 10^7^ trained or untrained splenocytes three days later. Tumor growth was compared at both 4 weeks and 10 weeks post-treatment. All seven mice injected with tumor cells only, developed tumors, along with six out of seven mice injected with tumor cells and either naïve splenocytes or splenocytes from Ag exposed mice. Most notable however, is that none of the mice injected with a single dose of 10^7^ trained splenocytes developed tumors at 4 weeks (**Figure 6**). Furthermore, tumor presence is scarcely detectable across all trained groups even at 10 weeks post tumor inoculation with a single dose of 10^7^ trained splenocytes (**Figure 6**). As greater than 80% of the trained slpenocytes are T cells, this observation underscores the potential therapeutic efficacy of trained T cells in the treatment of neuroblastoma.

### Inoculation of trained splenocytes did not induce autoimmune responses in cardiac tissue

**Figure 7:**
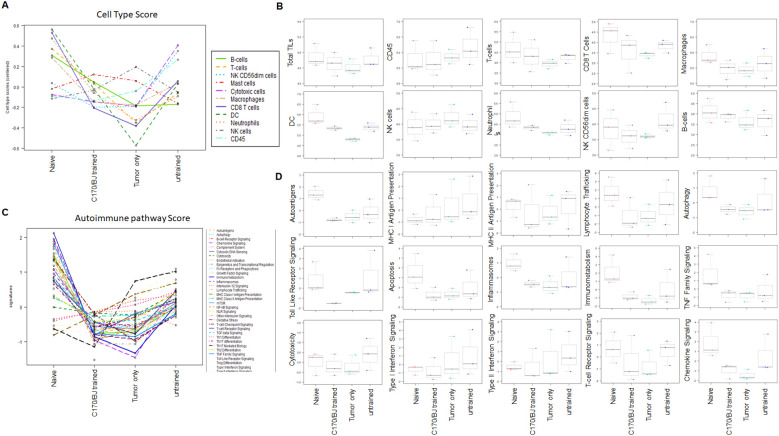
Lack of autoimmune response in cardiac tissue induced by inoculation of trained splenocytes. To investigate whether the inoculation of trained splenocytes induces autoimmune responses in cardiac tissue, antigen-exposed splenocytes were trained over 7 days with irradiated N2a cells treated with 1.5 µM C-170 in combination with 0.25 µM BET/JQ1. Subsequently, mice were intraperitoneally administered with 2x106 N2a cells along with 107 trained or untrained antigen-exposed splenocytes. Hearts were harvested at 4 weeks from naïve mice (n=3), mice with tumor only (n=3), tumor with untrained splenocytes (n=3), and tumor with trained splenocytes (n=3). The global expression of mRNA from the heart was investigated using NanoString Autoimmune Profiling arrays. **(A)** Trend dot-line plots of 11 immune cell types show that there is no significant increase in immune cell infiltration in the heart from mice administered with trained splenocytes compared with naïve mice. Cell type scores are calculated in log2 scale, an increase of 1 on the vertical axis corresponds to a doubling in abundance. **(B)** Box plots illustrate the distribution of immune cells by relative number present within the mouse heart, calculated by gene expression. The horizontal black line on the box plot represents the median expression, and each symbol represents a single individual. **(C)** Trend dot-line plots of immune pathways reveal no significant increase in immune activation in the heart from mice administered with trained splenocytes compared with naïve mice. **(D)** Immune pathway scores were presented as box plots for select immune pathways of interest. Results are expressed as mean score ± SD.

Previous studies from our laboratory revealed that a neuroblastoma vaccine approach incorporating BET/JQ1-treated cancer cells, along with anti-CTLA4 and anti-PDL-1 checkpoint inhibitors, resulted in potent anti-tumor immunity but also induced autoimmune responses and immune cell infiltration, most notably in cardiac tissue (35, 36). Thus, we aimed to investigate whether the administration of *ex vivo* trained splenocytes without checkpoint inhibitors also elicits autoimmune responses in the hearts of mice. We injected 2×10^6^ N2a cells and 1x10^7^ trained splenocytes intraperitoneally 3 days later in A/J mice. We harvested tissues from naïve mice (n=3), tumor only mice (n=3), tumor with untrained splenocytes (n=3) and tumor with trained splenocytes (n=3) after tumor and splenocyte inoculation at day 30 (n=12). The global expression of mRNA was investigated using NanoString mouse Autoimmune Profiling arrays. NanoString analysis revealed that there was no significant autoimmune response detected in cardiac tissue following administration of trained splenocytes in all the mice tested (**Figure 7**).

### The cytotoxicity of trained PBMCs/splenocytes relies on classical antigen-presenting cells

**Figure 8:**
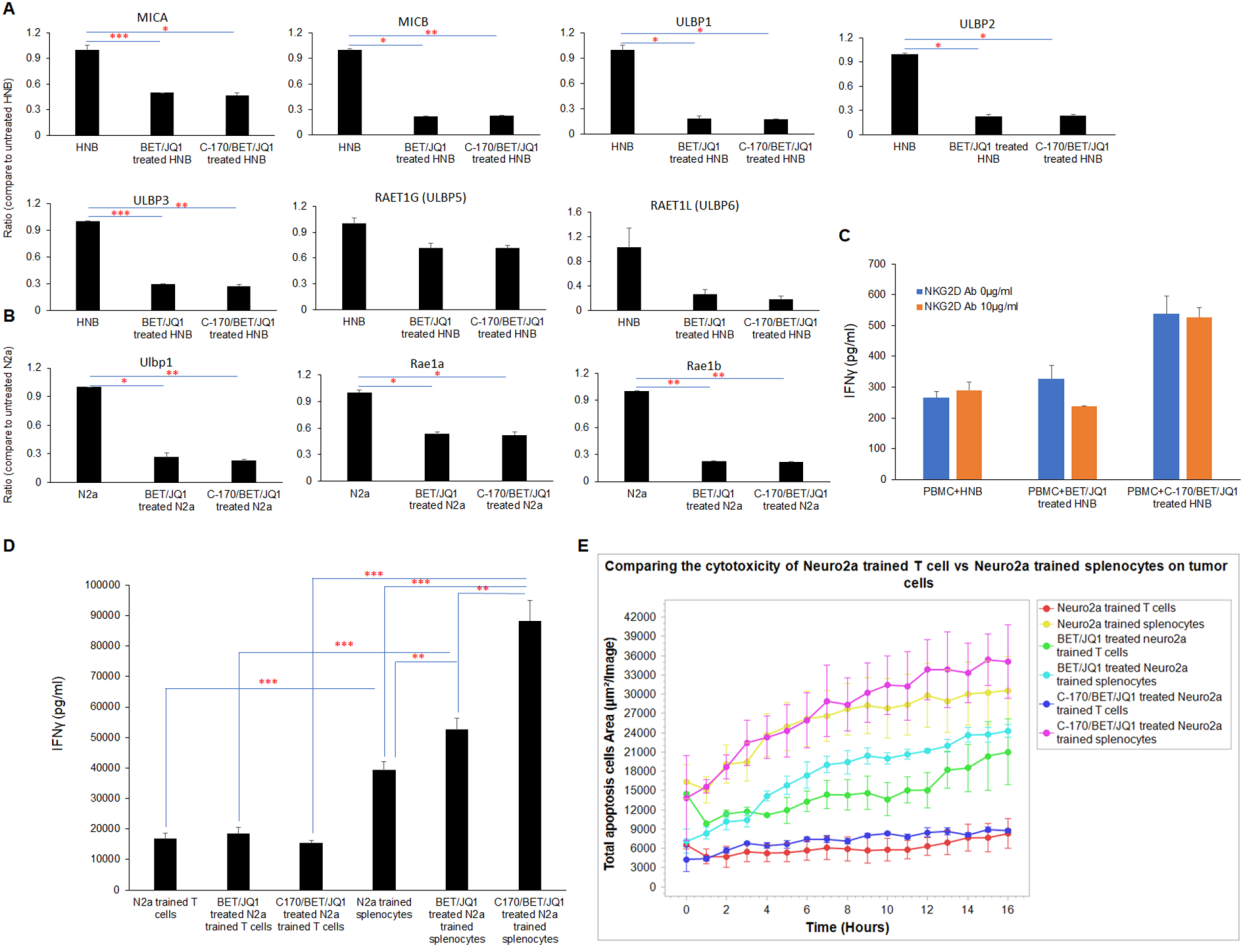
The cytotoxicity of trained PBMCs/splenocytes relies on classical antigen-presenting cells. **(A)** qRT-PCR) analysis of NKG2D ligands mRNA expression in HNB and **(B)** N2a cells treated with 0.25 µM BET/JQ1 or 0.25 µM BET/JQ1 combined with 1.5 µM C-170 for 3 and 4 days, utilizing GAPDH as an internal control. Results demonstrate significant downregulation of NKG2D ligands, including MICA, MICB, and ULPB1, 2, 3, 5, and 6 in HNB cells following treatment, with similar findings observed in mouse N2a treated cells (Ulbp1, Rae1a, and Rae1b). **(C)** Functional assessment of NKG2D in CD8+ T cell-mediated killing of HNB cells involved treatment with BET (0.25 μM) and JQ1 (0.25 μM), either alone or combined with 1.5 µM C-170 for 3 days, followed by irradiation (60 Gy). These irradiated treated (or untreated) HNB cells were then co-cultured with autologous PBMCs in the presence of 10μg/ml NKG2D-blocking antibody for 48 hours at effector-to-target cell ratios of 20:1. Results reveal that the anti-NKG2D antibody did not inhibit the production of IFNγ. **(D)** To determine if the anti-tumor immune response triggered by trained splenocytes depends on antigen-presenting cells, we selectively depleted dendritic cells, macrophages, NK cells and B cells from mouse splenocytes, leaving only the CD3+ T cell component. IFN-γ production between splenocytes and purified T cells trained with treated N2a tumor cells was quantified using ELISA assay. Results indicate significantly higher levels of IFN-γ production in the complete complement of splenocytes. **(E)** IncuCyte Live-Cell Analysis System was used to assess tumor cell cytotoxicity. Splenocytes and depleted T cells were trained with irradiated untreated N2a cells, N2a cells treated with 0.25µM BET/JQ1, or with 1.5 µM C-170 and 0.25 µM BET/JQ1 for 7 days. Subsequently, N2a cancer cells were co-cultured with untrained splenocytes and T cells or trained splenocytes and T cells at a ratio of 1:30 in 96-well plates. N2a tumor cells were stained with CytoLight Red dye, and apoptotic cells are marked by Caspase 3/7 Green. The yellow color indicates apoptotic tumor cells undergoing cytotoxicity. Target cell lysis by cytotoxic T cells (yellow color) was calculated based on the time-lapse images captured by the IncuCyte system over 21 hours, as depicted in the y-axis. Results demonstrate that the complete complement of splenocytes exhibit significantly enhanced cytotoxicity when compared to trained T cells devoid of APCs when both are co-cultured with N2a tumor cells. *p<0.05; **p<0.005; ***p<0.001.

The mechanism of training cytotoxic cells, could be a classic pathway dependent on antigen presentation via antigen presenting cells or through a non-classical pathway of T cell NKG2D ligand engagement on the tumor cells (42). To evaluate both mechanisms, we performed a series of studies evaluating both receptor ligands and activation studies. We observed a significant downregulation of NKG2D ligands, such as MICA, MICB and ULPB1, 2, and 3 following BET/JQ1treatment of the human tumor cells (**Figure 8**) with similar findings noted in mouse N2a treated cells as well (Ulbp1, Rae1a and Rae1b) (**Figure 8**). Subsequently, we examined the functional role of NKG2D in human CD8+ T cell-mediated killing of neuroblastoma cells through an *in vitro* IFNγ ELISA assay conducted in the presence of an NKG2D-blocking antibody. Our results revealed that the anti-NKG2D antibody did not inhibit the production of IFNγ (**Figure 8**). Together these findings suggest that the engagement of T cell NKG2D with non-classical NKG2D ligands (NKG2DLs) on tumor cells is not critical or at least necessary in mediating cytotoxicity of neuroblastoma cells.

To determine if the anti-tumor immune response triggered by trained splenocytes depends on antigen-presenting cells, we selectively depleted dendritic cells, macrophages, NK cells and B cells from mouse splenocytes, leaving only the CD3^+^ T cell component. We compared the production of IFN-γ and cell cytotoxicity between splenocytes and purified T cells trained with treated N2a tumor cells. Our results demonstrate that the complete compliment of splenocytes exhibit significantly higher levels of IFN-γ production (**Figure 8**) and enhanced cytotoxicity (**Figure 8**) when compared to trained T cells devoid of APCs in co-culture. These findings indicate that the classic pathway of antigen-presention via APCs plays a critical role in training T cells derived from splenocytes that are subsequently cytotoxic for the neuroblastoma tumor cells.

## Discussion

Pediatric solid tumors like neuroblastoma remain a major cause of illness and death, with traditional treatments offering limited success. This underscores the urgent need for innovative therapeutic strategies. Our research focuses on a novel immunotherapy approach, training autologous T cells derived from peripheral blood mononuclear cells (PBMCs) to harness their cytotoxic potential against neuroblastoma. This personalized therapy offers advantages, such as easy access to PBMCs, the ability to store cells long-term, and the potential for repeated treatments.

Previous studies show that neuroblastoma, especially in cases with MYCN amplification, is linked to immunosuppression. MYCN alters immune gene expression, reducing antigen presentation and immune cell function (31, 32, 34, 35). To address this, we use MYC inhibitors, such as I-BET726 and JQ1, which enhance tumor antigen presentation and immune activation (35). By combining MYC inhibition with small molecule treatments, we induce an immunogenic response in neuroblastoma cells, making them more recognizable and attackable by immune cells.

Our process involves co-culturing autologous PBMCs with irradiated neuroblastoma cells that have been treated with MYC inhibitors. This enhances PBMC training, leading to the production of cytotoxic T cells capable of targeting the tumor. The high levels of IFNγ secretion observed in the co-cultured cells indicate successful immune activation. Importantly, we found that combining MYC inhibition with STING pathway inhibition further enhanced immune response by downregulating DNA repair genes. This is significant because DNA repair deficiencies can make tumors more vulnerable to immune checkpoint inhibitors (41).

DNA repair involves several pathways, including mismatch repair (MMR), homology-dependent recombination (HR), and non-homologous end joining (NHEJ). Inhibiting MYC and STING led to downregulation of key genes in these pathways, such as MLH1, MSH2, and BRCA1, resulting in genomic instability. This instability generates neoantigens on the tumor surface, prompting an immune response (38). Additionally, DNA repair deficiencies lead to cytosolic DNA fragments, which activate the cGAS/STING pathway, triggering an inflammatory response and attracting immune cells to the tumor site (40, 41).

**Figure 9:**
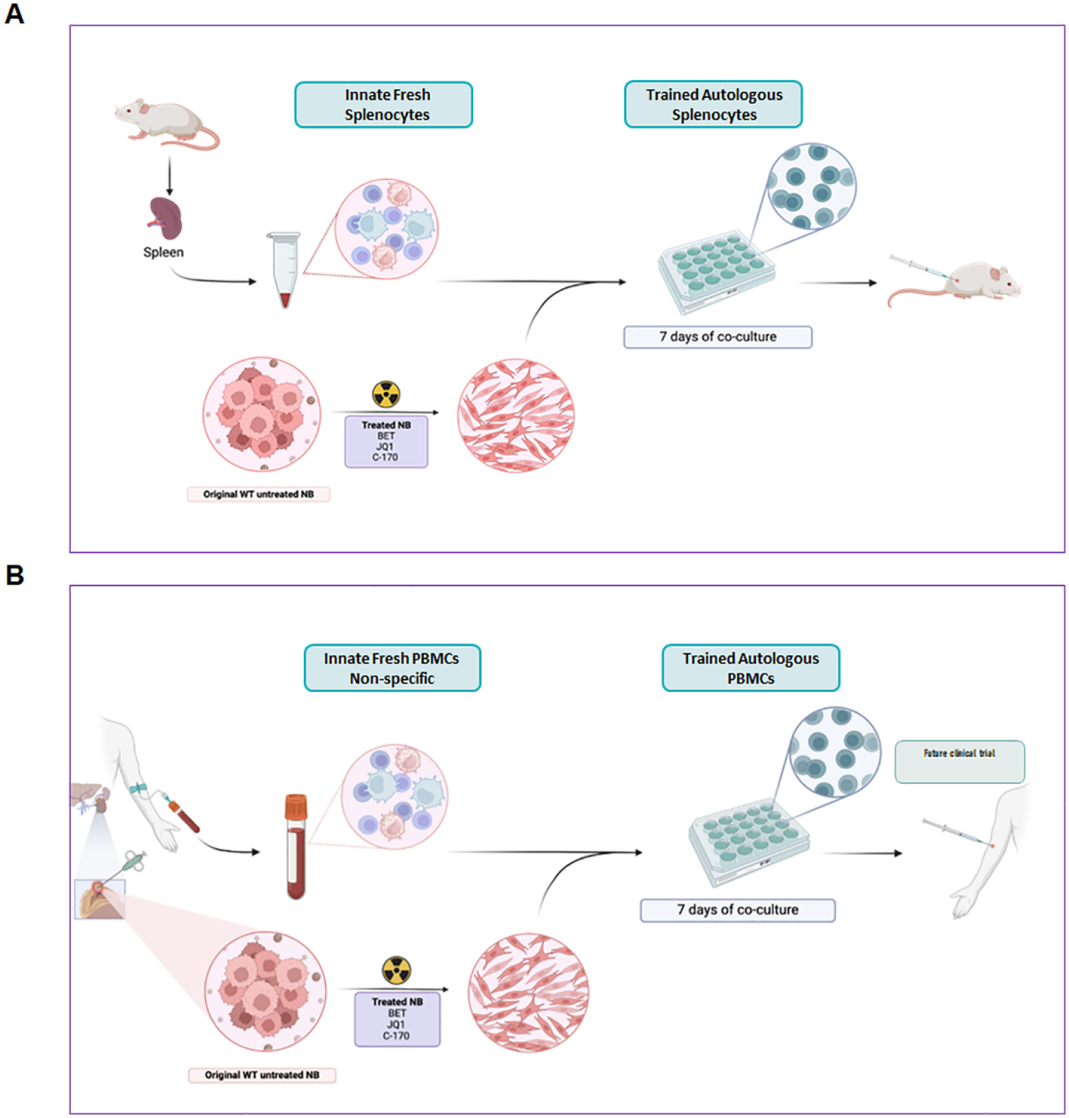
Graphic concept of trained autologous PBMCs and splenocytes for immunotherapy of neuroblastoma. Modulating tumor cells *in vitro* with small molecule inhibitors and irradiation produces an immunogenic phenotype that expands autologous splenocytes from mice **(A)** or PBMCs from human blood **(B)** into cytotoxic cells that are predominantly T cells. These autologous trained cells exhibit potent, targeted cytotoxicity against tumors in mice **(A)** and could potentially form the basis of adoptive cellular therapy in patients **(B)**.

*Ex vivo* training of PBMCs with treated neuroblastoma cells exhibited strong tumor-killing activity. Using the IncuCyte^®^ live-cell analysis system, we monitored real-time interactions between T cells and tumor cells, providing valuable insights into the cytotoxic process. In a neuroblastoma mouse model, reintroducing trained splenocytes led to tumor killing, demonstrating the potential of this approach for *in vivo* immunotherapy. Modulating tumor cells *in vitro* with small molecule inhibitors and radiation produces an immunogenic phenotype that expands autologous splenocytes from mice or PBMCs from human blood into cytotoxic T cells. These autologous trained splenocytes exhibit potent, targeted cytotoxicity against tumors in mice and could potentially form the basis of adoptive cellular therapy in patients (**Figure 9A**).

In contrast to traditional immune therapies that rely on antigen-specific CD8+ T cells, our method does not depend on NKG2D receptor-ligand interactions for immune synapse formation (42, 43). Instead, our results highlight the critical role of antigen-presenting cells (APCs) in facilitating T cell training. In our mouse model, splenocytes with APCs induced stronger IFNγ production and cytotoxicity against neuroblastoma cells than those without APCs.

As we have not performed direct, head-to-head comparisons with existing adoptive cellular therapies, our conclusions should be interpreted as preliminary and hypothesis-generating. We acknowledge the significant advances made with established modalities such as CAR-T and TCR-T cell therapies, which continue to demonstrate clinical promise. Rather than positioning our approach as a replacement or superior alternative, we propose it as a potential strategy—particularly in contexts where simplicity, speed, and safety are priorities. Our method leverages *ex vivo* tumor cell modification, which may mitigate systemic toxicities associated with MYC inhibition. It is technically straightforward, requiring only small tumor samples and peripheral blood mononuclear cells (PBMCs), and avoids the need for the complex genetic engineering intrinsic to CAR-T and TCR-T manufacturing. Additionally, because our approach is not limited to a few surface antigens, it may expand the repertoire of actionable tumor-associated targets. Nonetheless, we fully recognize that we have not yet performed formal comparative safety analyses against other immunotherapies, and further studies are essential to thoroughly assess both the safety and therapeutic potential of this strategy.

While our study shows promising results, further research is needed to optimize treatment protocols and ensure the balance between efficacy and safety. In our mouse model, high doses of trained T cells did not cause illness or immune activation in non-tumor tissues, indicating a favorable safety profile. However, more work is needed to refine the training process, including the optimal use of MYC inhibitors, the role of STING antagonists, and the durability of the trained PBMCs.

One critical area for future research is understanding the specific T cell component and receptors involved in recognizing tumor antigens that we have not as yet defined. We will also need to determine the longevity of the trained T cell response. This includes studying the memory and persistence of trained PBMCs to ensure lasting anti-tumor effects. Additionally, we need to explore the risk of T cell exhaustion, which could reduce the effectiveness of the therapy over time.

## Conclusion

Our study presents a promising form of autologous adoptive immunotherapy for pediatric solid tumors. By training autologous PBMCs against treated immunogenic neuroblastoma cells, we have demonstrated the potential for a personalized, effective, and apparently safe adoptive therapy. While further investigation is needed, our findings lay the groundwork for developing this approach into a viable therapy for neuroblastoma and possibly other solid tumors as well.

## Data Availability

The original contributions presented in the study are included in the article/**Supplementary Material**. Further inquiries can be directed to the corresponding author.

## References

[1] Du S Yan J Xue Y Zhong Y Dong Y. Adoptive cell therapy for cancer treatment. Explor (Beijing). (2023) 3:20210058. doi: 10.1002/EXP.20210058 PMC10624386 37933232

[2] Rosenberg SA Restifo NP. Adoptive cell transfer as personalized immunotherapy for human cancer. Science. (2015) 348:62–8. doi: 10.1126/science.aaa4967 PMC6295668 25838374

[3] Tesfaye M Savoldo B. Adoptive cell therapy in treating pediatric solid tumors. Curr Oncol Rep. (2018) 20:73. doi: 10.1007/s11912-018-0715-9 30069644 PMC6386156

[4] Guilhot F Roy L Martineua G Guilhot J Millot F. Immunotherapy in chronic myelogenous leukemia. Clin Lymphoma Myeloma. (2007) 7 Suppl 2:S64–70. doi: 10.3816/CLM.2007.s.004 17382015

[5] Aamir S Anwar MY Khalid F Khan SI Ali MA Khattak ZE. Systematic review and meta-analysis of CD19-specific CAR-T cell therapy in relapsed/refractory acute lymphoblastic leukemia in the pediatric and young adult population: safety and efficacy outcomes. Clin Lymphoma Myeloma Leuk. (2021) 21:e334–e47. doi: 10.1016/j.clml.2020.12.010 33573914

[6] Timmers M Roex G Wang Y Campillo-Davo D Van Tendeloo VFI Chu Y. Chimeric antigen receptor-modified T cell therapy in multiple myeloma: beyond B cell maturation antigen. Front Immunol. (2019) 10:1613. doi: 10.3389/fimmu.2019.01613 31379824 PMC6646459

[7] Rivoltini L Arienti F Orazi A Cefalo G Gasparini M Gambacorti-Passerini C. Phenotypic and functional analysis of lymphocytes infiltrating paediatric tumours, with a characterization of the tumour phenotype. Cancer Immunol Immunother. (1992) 34:241–51. doi: 10.1007/BF01741792 PMC11038025 1311218

[8] Zemanek T Nova Z Nicodemou A. Tumor-infiltrating lymphocytes and adoptive cell therapy: state of the art in colorectal, breast and lung cancer. Physiol Res. (2023) 72:S209–S24. doi: 10.33549/physiolres PMC10669950 37888965

[9] Hensel J Metts J Gupta A Ladle BH Pilon-Thomas S Mullinax J. Adoptive cellular therapy for pediatric solid tumors: beyond chimeric antigen receptor-T cell therapy. Cancer J. (2022) 28:322–7. doi: 10.1097/PPO.0000000000000603 PMC9847472 35880942

[10] Richards RM Sotillo E Majzner RG. CAR T cell therapy for neuroblastoma. Front Immunol. (2018) 9:2380. doi: 10.3389/fimmu.2018.02380 30459759 PMC6232778

[11] Seidel D Shibina A Siebert N Wels WS Reynolds CP Huebener N. Disialoganglioside-specific human natural killer cells are effective against drug-resistant neuroblastoma. Cancer Immunol Immunother. (2015) 64:621–34. doi: 10.1007/s00262-015-1669-5 PMC11029162 25711293

[12] Majzner RG Mackall CL. Tumor antigen escape from CAR T-cell therapy. Cancer Discov. (2018) 8:1219–26. doi: 10.1158/2159-8290.CD-18-0442 30135176

[13] Louis CU Savoldo B Dotti G Pule M Yvon E Myers GD. Antitumor activity and long-term fate of chimeric antigen receptor-positive T cells in patients with neuroblastoma. Blood. (2011) 118:6050–6. doi: 10.1182/blood-2011-05-354449 PMC3234664 21984804

[14] Sterner RC Sterner RM. CAR-T cell therapy: current limitations and potential strategies. Blood Cancer J. (2021) 11:69. doi: 10.1038/s41408-021-00459-7 33824268 PMC8024391

[15] Moradi V Omidkhoda A Ahmadbeigi N. The paths and challenges of “off-the-shelf” CAR-T cell therapy: An overview of clinical trials. BioMed Pharmacother. (2023) 169:115888. doi: 10.1016/j.biopha.2023.115888 37979380

[16] Newick K O’Brien S Moon E Albelda SM. CAR T cell therapy for solid tumors. Annu Rev Med. (2017) 68:139–52. doi: 10.1146/annurev-med-062315-120245 27860544

[17] Neelapu SS Tummala S Kebriaei P Wierda W Gutierrez C Locke FL. Chimeric antigen receptor T-cell therapy - assessment and management of toxicities. Nat Rev Clin Oncol. (2018) 15:47–62. doi: 10.1038/nrclinonc.2017.148 28925994 PMC6733403

[18] Frey N Porter D. Cytokine release syndrome with chimeric antigen receptor T cell therapy. Biol Blood Marrow Transplant. (2019) 25:e123–e7. doi: 10.1016/j.bbmt.2018.12.756 30586620

[19] Hay KA. Cytokine release syndrome and neurotoxicity after CD19 chimeric antigen receptor-modified (CAR-) T cell therapy. Br J Haematol. (2018) 183:364–74. doi: 10.1111/bjh.2018.183.issue-3 30407609

[20] Freyer CW Porter DL. Cytokine release syndrome and neurotoxicity following CAR T-cell therapy for hematologic Malignancies. J Allergy Clin Immunol. (2020) 146:940–8. doi: 10.1016/j.jaci.2020.07.025 32771558

[21] Dagar G Gupta A Masoodi T Nisar S Merhi M Hashem S. Harnessing the potential of CAR-T cell therapy: progress, challenges, and future directions in hematological and solid tumor treatments. J Transl Med. (2023) 21:449. doi: 10.1186/s12967-023-04292-3 37420216 PMC10327392

[22] Xiao X Huang S Chen S Wang Y Sun Q Xu X. Mechanisms of cytokine release syndrome and neurotoxicity of CAR T-cell therapy and associated prevention and management strategies. J Exp Clin Cancer Res. (2021) 40:367. doi: 10.1186/s13046-021-02148-6 34794490 PMC8600921

[23] Velasco R Mussetti A Villagran-Garcia M Sureda A. CAR T-cell-associated neurotoxicity in central nervous system hematologic disease: Is it still a concern? Front Neurol. (2023) 14:1144414. doi: 10.3389/fneur.2023.1144414 37090983 PMC10117964

[24] Kobayashi H Tanaka Y Yagi J Minato N Tanabe K. Phase I/II study of adoptive transfer of gammadelta T cells in combination with zoledronic acid and IL-2 to patients with advanced renal cell carcinoma. Cancer Immunol Immunother. (2011) 60:1075–84. doi: 10.1007/s00262-011-1021-7 PMC11029699 21519826

[25] Meraviglia S Eberl M Vermijlen D Todaro M Buccheri S Cicero G. *In vivo* manipulation of Vgamma9Vdelta2 T cells with zoledronate and low-dose interleukin-2 for immunotherapy of advanced breast cancer patients. Clin Exp Immunol. (2010) 161:290–7. doi: 10.1111/j.1365-2249.2010.04167.x PMC2909411 20491785

[26] Deniger DC Maiti SN Mi T Switzer KC Ramachandran V Hurton LV. Activating and propagating polyclonal gamma delta T cells with broad specificity for Malignancies. Clin Cancer Res. (2014) 20:5708–19. doi: 10.1158/1078-0432.CCR-13-3451 PMC4233015 24833662

[27] Razmara AM Farley LE Harris RM Judge SJ Lammers M Iranpur KR. Preclinical evaluation and first-in-dog clinical trials of PBMC-expanded natural killer cells for adoptive immunotherapy in dogs with cancer. J Immunother Cancer. (2024) 12(4):e007963. doi: 10.1136/jitc-2023-007963 38631708 PMC11029326

[28] Vardam-Kaur T Pathangey LB McCormick DJ Bergsagel PL Cohen PA Gendler SJ. Multipeptide stimulated PBMCs generate T(EM)/T(CM) for adoptive cell therapy in multiple myeloma. Oncotarget. (2021) 12:2051–67. doi: 10.18632/oncotarget.v12i20 PMC8487724 34611479

[29] Chapuis AG Roberts IM Thompson JA Margolin KA Bhatia S Lee SM. T-cell therapy using interleukin-21-primed cytotoxic T-cell lymphocytes combined with cytotoxic T-cell lymphocyte antigen-4 blockade results in long-term cell persistence and durable tumor regression. J Clin Oncol. (2016) 34:3787–95. doi: 10.1200/JCO.2015.65.5142 PMC5477923 27269940

[30] Dang CV. MYC on the path to cancer. Cell. (2012) 149:22–35. doi: 10.1016/j.cell.2012.03.003 22464321 PMC3345192

[31] Layer JP Kronmuller MT Quast T van den Boorn-Konijnenberg D Effern M Hinze D. Amplification of N-Myc is associated with a T-cell-poor microenvironment in metastatic neuroblastoma restraining interferon pathway activity and chemokine expression. Oncoimmunology. (2017) 6:e1320626. doi: 10.1080/2162402X.2017.1320626 28680756 PMC5486176

[32] Casey SC Baylot V Felsher DW. The MYC oncogene is a global regulator of the immune response. Blood. (2018) 131:2007–15. doi: 10.1182/blood-2017-11-742577 PMC5934797 29514782

[33] Zimmerli D Brambillasca CS Talens F Bhin J Linstra R Romanens L. MYC promotes immune-suppression in triple-negative breast cancer via inhibition of interferon signaling. Nat Commun. (2022) 13:6579. doi: 10.1038/s41467-022-34000-6 36323660 PMC9630413

[34] Zhang P Wu X Basu M Dong C Zheng P Liu Y. MYCN amplification is associated with repressed cellular immunity in neuroblastoma: an in silico immunological analysis of TARGET database. Front Immunol. (2017) 8:1473. doi: 10.3389/fimmu.2017.01473 29163537 PMC5675839

[35] Wu X Nelson M Basu M Srinivasan P Lazarski C Zhang P. MYC oncogene is associated with suppression of tumor immunity and targeting Myc induces tumor cell immunogenicity for therapeutic whole cell vaccination. J Immunother Cancer. (2021) 9(3):e001388. doi: 10.1136/jitc-2020-001388 33757986 PMC7993333

[36] Wu X Srinivasan P Basu M Zhang P Saruwatari M Thommandru B. Tumor Apolipoprotein E is a key checkpoint blocking anti-tumor immunity in mouse melanoma. Front Immunol. (2022) 13:991790. doi: 10.3389/fimmu.2022.991790 36341364 PMC9626815

[37] Kilkenny C Browne WJ Cuthill IC Emerson M Altman DG. Improving bioscience research reporting: the ARRIVE guidelines for reporting animal research. PLoS Biol. (2010) 8:e1000412. doi: 10.1371/journal.pbio.1000412 20613859 PMC2893951

[38] Zhang J Shih DJH Lin SY. Role of DNA repair defects in predicting immunotherapy response. Biomark Res. (2020) 8:23. doi: 10.1186/s40364-020-00202-7 32612833 PMC7325270

[39] Xu Y Nowsheen S Deng M. DNA repair deficiency regulates immunity response in cancers: molecular mechanism and approaches for combining immunotherapy. Cancers (Basel). (2023) 15(5):1619. doi: 10.3390/cancers15051619 36900418 PMC10000854

[40] Cimprich KA Li GM Demaria S Gekara NO Zha S Chen Q. The crosstalk between DNA repair and immune responses. Mol Cell. (2023) 83:3582–7. doi: 10.1016/j.molcel.2023.09.022 37863025

[41] Barros EM McIntosh SA Savage KI. The DNA damage induced immune response: Implications for cancer therapy. DNA Repair (Amst). (2022) 120:103409. doi: 10.1016/j.dnarep.2022.103409 36308822

[42] Lerner EC Woroniecka KI D’Anniballe VM Wilkinson DS Mohan AA Lorrey SJ. CD8(+) T cells maintain killing of MHC-I-negative tumor cells through the NKG2D-NKG2DL axis. Nat Cancer. (2023) 4:1258–72. doi: 10.1038/s43018-023-00600-4 PMC10518253 37537301

[43] Markiewicz MA Carayannopoulos LN Naidenko OV Matsui K Burack WR Wise EL. Costimulation through NKG2D enhances murine CD8+ CTL function: similarities and differences between NKG2D and CD28 costimulation. J Immunol. (2005) 175:2825–33. doi: 10.4049/jimmunol.175.5.2825 16116168

