# Supplementary material for: Trained autologous cytotoxic T-cells derived from PBMCs or splenocytes for immunotherapy of neuroblastoma
Source: Front Immunol. 2025 Jun 9;16:1546441. doi: 10.3389/fimmu.2025.1546441 (PMC12183212; doi:10.3389/fimmu.2025.1546441)
Supplement: Supplementary file 1 [file DataSheet1.pdf]

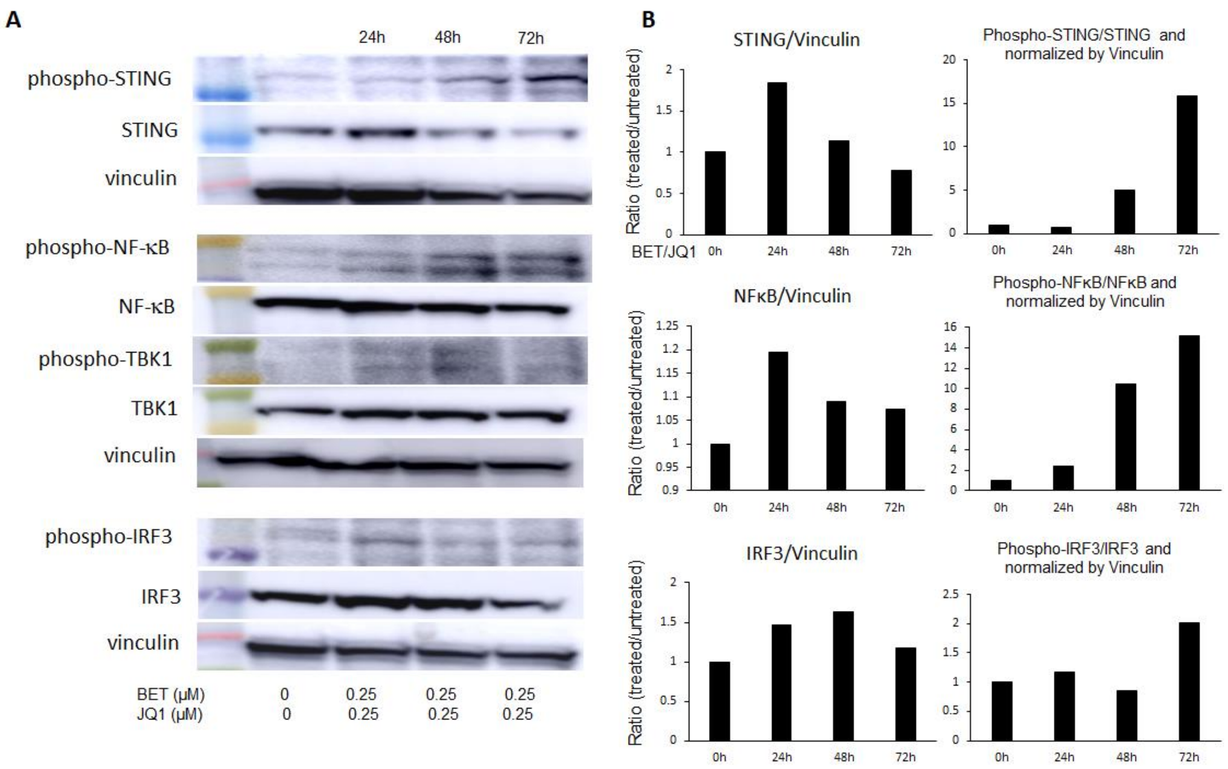

Supplementary Figure 1. Western blot analysis illustrating (A) the phosphorylation of STING, NF-κB, TBK1, and IRF-3 in the cGAS–STING pathway in HNB-1 cells following treatment with BET (0.25 μM) and JQ1 (0.25 μM) for three days. Vinculin served as a loading control. **(B)** Semi-quantitative analysis of Western blot results using ImageJ.

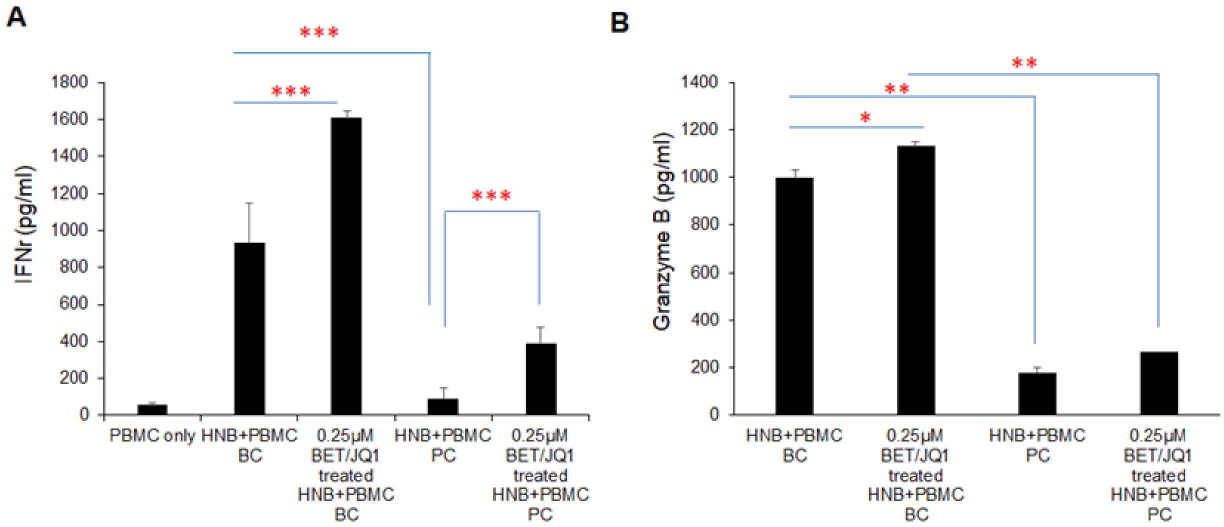

Supplement Figure 1. PBMCs collected from patients before chemotherapy exhibited a more robust response to MYC inhibited tumor cells compared to those collected after chemotherapy. HNB cells were treated with 0.25 μM BET and 0.25 μM JQ1 for 3 days, followed by irradiation (60 Gy). Subsequently, these treated or untreated HNB cells were co-cultured with autologous PBMCs collected before chemotherapy (PBMC BC) or after chemotherapy (PBMC PC) for 48 hours at effector-to-target cell ratios of 20:1. **(A)** IFNγ production and **(B)** Granzyme B production from PBMCs in the tumor cell-PBMC reaction was assessed using ELISA. Results revealed a significant increase in IFNγ and Granzyme B concentrations in the media when PBMCs collected before chemotherapy were cocultured with treated HNB cells. Data presented are representative of three independent experiments and shown as the mean ± SD. Statistical significance was determined by unpaired two-tailed Student's t-test (*p < 0.05; **p < 0.01, ***p < 0.001).

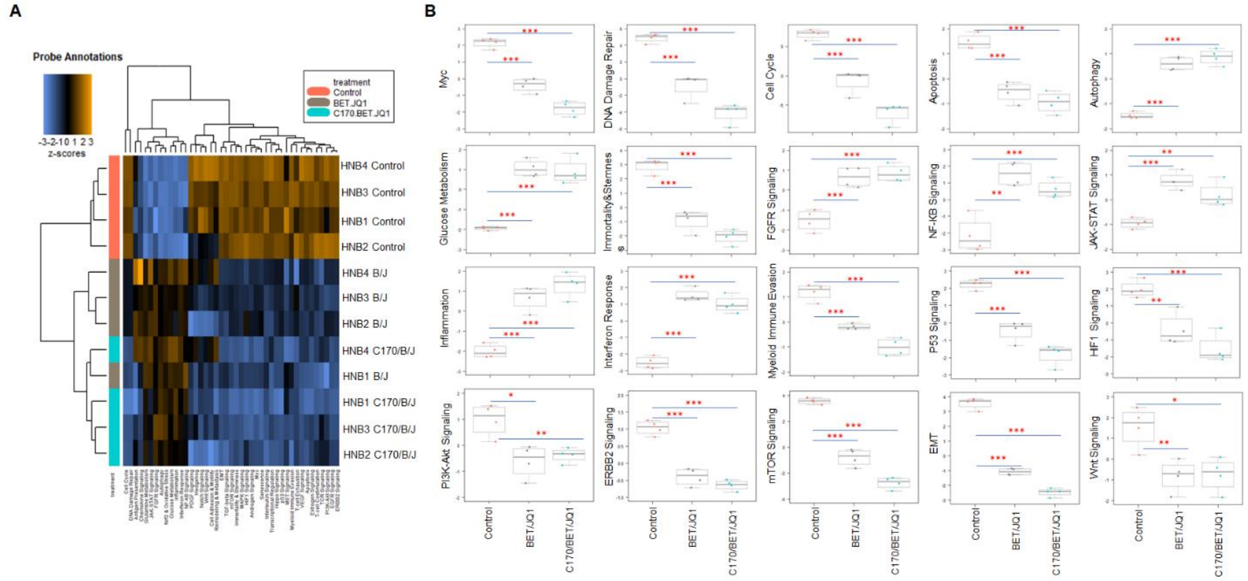

Supplement Figure 3. Modulation of cancer signaling pathways in human neuroblastoma cells through Myc targeting and Sting inhibition. HNB1-4 cells were treated with either 0.25 μM BET and 0.25 μM JQ1 or 1.5 μM C-170 and 0.25 μM BET/JQ1 for a duration of 3 days. Subsequently, gene expression profiles were analyzed using NanoString Human Cancer Signaling 360 Profiling. **(A)** A heat map depicting pathway scores was generated, with pathways listed on the horizontal axis and samples on the vertical axis. Pathways exhibiting elevated scores are highlighted in orange, while those with lower scores are shown in blue. Notably, distinct separation of pathway activity is observed among cells treated with 0.25 μM BET/0.25 μM JQ1 (n=4), 1.5 μM C-170/0.25 μM BET/0.25 μM JQ1 (n=4), and untreated cells (n=4). **(B)** Box plots were utilized to illustrate significantly altered pathway scores in treated versus untreated cells, including median expression levels. The upper and lower error bars represent the maximum and minimum expression levels, respectively. Statistical analysis was performed using an unpaired two-tailed Student's t-test (*p < 0.05; **p < 0.01; ***p < 0.001).

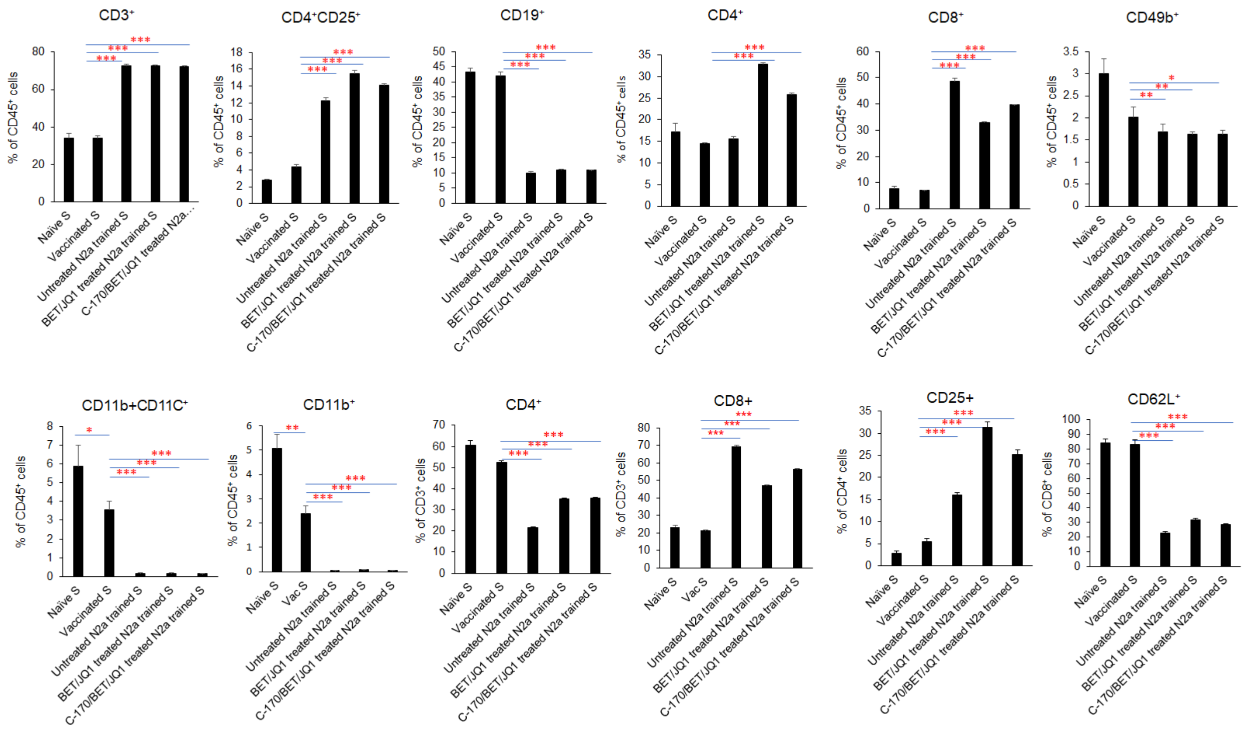

Supplement Figure 4. The flow cytometry analysis of the splenocytes trained with treated N2a tumor cells. Splenocytes were trained over 7 days with irradiated wild-type N2a cells, N2a cells treated with 0.25μM BET/JQ1, or N2a cells treated with 1.5 μM C-170 in combination with 0.25 μM BET/JQ1. Following training, splenocytes were stained with specific mAbs and analyzed by flow cytometry. The relevant isotype control sample was set as negative control (data not shown). Data are representative of three independent experiments. The bars represent means±SD. Statistical significance was determined using unpaired two-tailed Student's t-test (*p < 0.05, **p < 0.01, ***p < 0.001), (n=3 tests for each group).
