# Supplementary material for: Trained autologous cytotoxic T-cells derived from PBMCs or splenocytes for immunotherapy of neuroblastoma
Source: Front Immunol. 2025 Jun 9;16:1546441. doi: 10.3389/fimmu.2025.1546441 (PMC12183212; doi:10.3389/fimmu.2025.1546441)
Supplement: Supplementary file 2 [file Presentation1.pptx]

## Slide 1
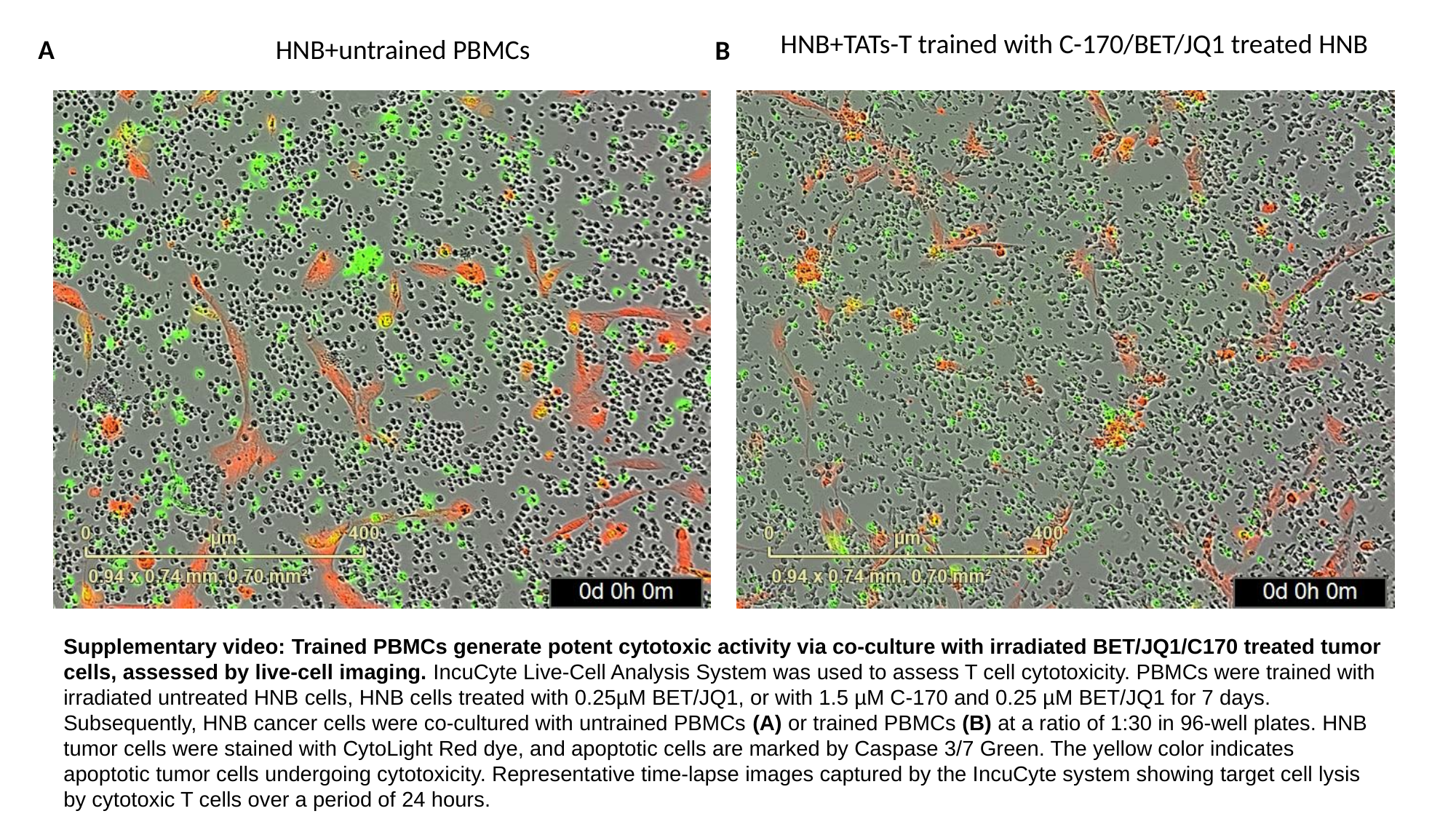

HNB+TATs-T trained with C-170/BET/JQ1 treated HNB
A
HNB+untrained PBMCs
B
Supplementary video: Trained PBMCs generate potent cytotoxic activity via co-culture with irradiated BET/JQ1/C170 treated tumor cells, assessed by live-cell imaging. IncuCyte Live-Cell Analysis System was used to assess T cell cytotoxicity. PBMCs were trained with irradiated untreated HNB cells, HNB cells treated with 0.25µM BET/JQ1, or with 1.5 µM C-170 and 0.25 µM BET/JQ1 for 7 days. Subsequently, HNB cancer cells were co-cultured with untrained PBMCs (A) or trained PBMCs (B) at a ratio of 1:30 in 96-well plates. HNB tumor cells were stained with CytoLight Red dye, and apoptotic cells are marked by Caspase 3/7 Green. The yellow color indicates apoptotic tumor cells undergoing cytotoxicity. Representative time-lapse images captured by the IncuCyte system showing target cell lysis by cytotoxic T cells over a period of 24 hours.
